# Characterization of pathogenesis of and immune response to *Burkholderia pseudomallei* K96243 using both inhalational and intraperitoneal infection models in BALB/c and C57BL/6 mice

**DOI:** 10.1371/journal.pone.0172627

**Published:** 2017-02-24

**Authors:** Jeremy J. Bearss, Melissa Hunter, Jennifer L. Dankmeyer, Kristen A. Fritts, Christopher P. Klimko, Chris H. Weaver, Jennifer L. Shoe, Avery V. Quirk, Ronald G. Toothman, Wendy M. Webster, David P. Fetterer, Joel A. Bozue, Patricia L. Worsham, Susan L. Welkos, Kei Amemiya, Christopher K. Cote

**Affiliations:** 1 Pathology Division, United States Army Medical Research Institute of Infectious Diseases (USAMRIID), Fort Detrick, Frederick, MD, United States of America; 2 Bacteriology Division, USAMRIID, Fort Detrick, Frederick, MD, United States of America; 3 BioStatisitics Division, USAMRIID, Fort Detrick, Frederick, MD, United States of America; University of Toledo College of Medicine and Life Sciences, UNITED STATES

## Abstract

*Burkholderia pseudomallei*, the etiologic agent of melioidosis, is a Gram negative bacterium designated as a Tier 1 threat. This bacterium is known to be endemic in Southeast Asia and Northern Australia and can infect humans and animals by several routes. Inhalational melioidosis has been associated with monsoonal rains in endemic areas and is also a significant concern in the biodefense community. There are currently no effective vaccines for *B*. *pseudomallei* and antibiotic treatment can be hampered by non-specific symptomology and also the high rate of naturally occurring antibiotic resistant strains. Well-characterized animal models will be essential when selecting novel medical countermeasures for evaluation prior to human clinical trials. Here, we further characterize differences between the responses of BALB/c and C57BL/6 mice when challenged with low doses of a low-passage and well-defined stock of *B*. *pseudomallei* K96243 via either intraperitoneal or aerosol routes of exposure. Before challenge, mice were implanted with a transponder to collect body temperature readings, and daily body weights were also recorded. Mice were euthanized on select days for pathological analyses and determination of the bacterial burden in selected tissues (blood, lungs, liver, and spleen). Additionally, spleen homogenate and sera samples were analyzed to better characterize the host immune response after infection with aerosolized bacteria. These clinical, pathological, and immunological data highlighted and confirmed important similarities and differences between these murine models and exposure routes.

## Introduction

*Burkholderia pseudomallei* is the causative agent of melioidosis and is categorized as a Tier 1 biological select agent by the U.S. Department of Health and Human Services [1]. It is a Gram negative bacillus that is commonly found in soil and water in Northern Australia and Thailand and is a known cause of sepsis [2–7]. There is recent evidence to support the concept that *B*. *pseudomallei* may be distributed in tropical locations located throughout the world [8–14]. Melioidosis is commonly initiated from the introduction of the bacterium into a subcutaneous injury. *B*. *pseudomallei* can cause either acute sepsis or a chronic infection [9, 15–18]. Acute sepsis generally manifests within 1 to 21 days, while a chronic infection with *B*. *pseudomallei* is characterized by symptoms that last substantially longer (i.e. greater than two months). There are currently no effective vaccines for melioidosis [19], and treatment can be hampered by non-specific symptomology, high frequencies of naturally occurring antibiotic resistance, and the propensity of the bacterium to cause a chronic infection that emerges years (to decades) later [20, 21]. Several factors, including agricultural occupational exposure in endemic areas, alcoholism, or diabetes mellitus have been shown to be important risk factors for presenting with melioidosis [2, 22, 23]. Of specific concern to the biodefense research community is the fact that *B*. *pseudomallei* is known to be transmitted to humans via inhalation, most often associated with strong rains and winds in geographic areas where the bacterium is endemic [7, 24, 25].

There has been significant effort invested in developing appropriate animal models of melioidosis (i.e. mice, rats, hamsters, goats, and non-human primates) [26–30]. Small animal models, specifically the BALB/c and C57BL/6 mouse models, have been used to mimic both the acute and chronic stages of *B*. *pseudomallei* infection [23, 27, 31–39]. The BALB/c mouse model results in an acute infection after either intraperitoneal (IP) injection or aerosol exposure [27, 36, 37, 40]; while C57BL/6 mice are considerably more resistant to acute infection and hypothesized to be a suitable model for chronic infection after exposure by either IP injection or by a pulmonary route [27, 37, 40, 41]. The BALB/c mouse model is a useful tool in identifying the mechanisms of virulence of *B*. *pseudomallei*, as well as for preliminary screening for vaccine or therapeutic efficacy [27, 31, 35, 36, 42–45]. The disease model is, of course, dependent upon the different routes of infection used in these studies (i.e. IP or intranasal/inhalation) and may be dependent upon the *B*. *pseudomallei* strain used for challenges [36, 45, 46]. These routes of exposure using BALB/c mice result in an acute disseminated infection that mimics some of the features of human melioidosis. BALB/c mice develop numerous abscesses and/or pyogranulomatous masses in various organs or locations throughout the body (i.e. spleen, liver, lungs) [36]. Depending upon the dose administered, BALB/c mice can succumb to infection within 2 to 3 days post exposure. The C57BL/6 mice generally clear the initial bacterial challenge (unless large doses are delivered) to below the limits of detection in both the spleen and liver within days to weeks of being exposed to the bacterium [33, 34]. It has been reported that C57BL/6 mice may remain asymptomatic for months before spontaneous disease occurs [33]. The spontaneous activation appears in the form of localized pyogranulomatous lesions (i.e. lesions on the ear, tail, liver and spleen). The long term latency of the infection in C57BL/6 mice potentially mimics that of the chronic human illness. It is important to note that *B*. *pseudomallei* is a facultative intracellular pathogen and its ability to survive within host cells may be an important aspect of the disease chronicity [47, 48].

The formation of multinucleated giant cells (MNGCs) by infected host cells has been well documented using *in vitro* assays with macrophage-like cell culture lines infected with *Burkholderia* species [36, 49–53], primary mouse macrophages [54], and nonphagocytic cell lines [52, 53]. MNGCs, referred to as a “hallmark” of *B*. *pseudomallei* infection [55], have been reported in other studies of chronic melioidosis in mice [33, 55], Madagascar hissing cockroaches [56], and in human autopsies [57]. Surprisingly, there are few descriptions of MNGCs in mice infected with *B*. *pseudomallei* [33, 55].

Since mouse models will be essential for preliminary prescreening and subsequent down selection of novel medical countermeasures (i.e. therapeutics, vaccines, or combination regimens); a better characterization of the extent and significance of MNGCs in mice is warranted. This report adds to the growing body of literature characterizing the murine experimental models of melioidosis. We described results of a comparison between BALB/c and C57BL/6 mice challenged with either an IP injection or by exposure to aerosolized bacteria using the *B*. *pseudomallei* strain K96243. In both cases, the challenge doses were purposefully low to more fully characterize the disease progression and to look for signs of chronic infection. To depict a more complete disease model, weight and temperatures were recorded daily, bacterial burdens were determined and immunological and histological analyses are reported.

## Materials and methods

### Animal challenges

Groups of 80 BALB/c or C57BL/6 mice (Charles River-Frederick, MD; female 7–10 weeks of age at time of exposure to bacteria) were challenged by the IP or inhalational route with a low passage and well-defined stock of *B*. *pseudomallei* K96243 [36, 58]. The bacteria used were grown in 4% glycerol (Sigma Aldrich, St. Louis, MO)-1% tryptone (Difco, Becton Dickinson, Sparks, MD) and 5% NaCl (Sigma Aldrich, St. Louis, MO) broth (GTB) at 37° C with shaking at 200 rpm and were harvested from a late log phase culture. The bacteria were resuspended in GTB and quantified via OD_620_ estimations. The actual delivered doses of bacteria were then verified by plate counts on sheep’s blood agar (SBA) plates (Remel, ThermoFisher Scientific, Waltham, MA). Each IP dose was delivered in 200 μl of GTB medium. The IP challenge groups doses were as follows: BALB/c mice received approximately 3.0x10^4^ colony forming units (CFU) (approximately 0.49 LD_50_ equivalent based upon previous day 21 LD_50_ calculations) and C57BL/6 mice received approximately 9.2x10^5^ CFU (approximately 0.42 LD_50_ equivalents based upon previous day 21 LD_50_ calculations) [36, 41]. Exposure to aerosolized bacteria was accomplished as previously described [59]. Briefly, mice were transferred to wire mesh cages (up to 10 mice per cage) and up to four wire mesh cages were placed in a whole-body aerosol chamber within a class three biological safety cabinet located inside a BSL-3 laboratory. Mice were exposed to aerosolized *B*. *pseudomallei* strain K96243 created by a three-jet collision nebulizer. Samples were collected from the all-glass impinger (AGI) and analyzed by performing CFU calculations to determine the inhaled dose of *B*. *pseudomallei*. The inhalational challenge doses were as follows: BALB/c mice received approximately 5 CFU (approximately 0.2 LD_50_ equivalents based upon previous day 21 LD_50_ calculations) and C57BL/6 mice received approximately 18 CFU (approximately 0.05 LD_50_ equivalents based upon previously calculated day 21 LD_50_ calculations) (Waag and Soffler, personal communication).

Prior to challenge BALB/c and C57BL/6 female mice were implanted subcutaneously with Electronic ID Transponder–IPTT 300 (Bio Medic Data Systems-BMDS, Seaford Delaware). Mice were scanned for daily temperatures via Smart Probe SP-6005 (BMDS, Seaford, Delaware) and daily weights were determined on Adventurer Pro Balance (Ohaus, Pasippany, NJ). The daily readings represented all mice surviving on that day. We chose to maximize our data set in this manner because of the diverse nature of *B*. *pseudomallei* infections, particularly those initiated by low concentrations of bacteria. These data were recorded by host DAS-8001 Data Acquisition System (BMDS) and stored in Excel format. Mice were monitored for clinical signs and symptoms for 59 days for the IP challenge group and 91 days for the inhalational challenge group. Early endpoint euthanasia was employed by CO_2_ exposure in a uniform manner to limit pain and distress of the mice. For dissemination studies following IP challenge, mice were euthanized by exsanguination under deep anesthesia on days 0 (approximately 4–6 hours post exposure to *B*. *pseudomallei*), 2, 4, 7, 15, 22, and 28 post-infection, and lungs, spleen, and liver samples were collected. On day 59 survivors were euthanized and only their spleens were examined for bacterial burden. For dissemination studies following exposure to aerosolized *B*. *pseudomallei*, mice were euthanized by exsanguination under deep anesthesia on days 0 (approximately 4–6 hours post exposure to *B*. *pseudomallei*), 2, 4, 7, 15, 22, 28, 59, and 91 days post-infection and lungs, spleen, and liver samples were collected. Only C57BL/6 mice remained for sampling beyond day 22. Tissues were harvested, weighed, homogenized, and then CFU were enumerated on SBA plates. The limit of detection for spleen, liver, and lungs was approximately 10 CFU/ml. Due to blood volume constrains, the limit of detection for blood was approximately 100 CFU/ml. Confirmatory bacterial identification was also performed using *Burkholderia cepacia* selective agar plates (Remel, ThermoFisher Scientific, Waltham, MA). The surviving C57BL/6 mice in the inhalational challenge group were retained through day 91 in an attempt to identify any signs of chronicity (i.e. clinical signs, such as weight loss, temperature increase, altered appearance, or bacterial burden in tissues after euthanasia).

### Ethics statement

Animal research at the United States Army Medical Research Institute of Infectious Diseases (USAMRIID) was conducted under an animal use protocol approved by the USAMRIID Institutional Animal Care and Use Committee (IACUC) in compliance with the Animal Welfare Act, PHS Policy, and other Federal statutes and regulations relating to animals and experiments involving animals. The facility where this research was conducted is accredited by the Association for Assessment and Accreditation of Laboratory Animal Care International (AAALACi) and adheres to principles stated in the Guide for the Care and Use of Laboratory Animals (National Research Council, 2011). Challenged mice were observed at least daily for up to 91 days for clinical signs of illness. Early interventions endpoints were used during all studies and mice were humanely euthanized when moribund, according to an endpoint score sheet. Animals were scored on a scale of 0–11: 0–2 = no significant clinical signs (e.g., slightly ruffled fur); 3–7 = significant clinical symptoms such as subdued behavior, hunched appearance, absence of grooming, hind limb issues of varying severity and/or pyogranulomatous swelling of varying severity (increased monitoring was warranted); 8–11 = distress. Those animals receiving a score of 8–11 were humanely euthanized. However, even with multiple observations per day, some animals died as a direct result of the infection.

### Histological pathology

Post-mortem tissues were collected from euthanized mice and fixed in 10% neutral buffered formalin for ≥ 21 days. Samples were embedded in paraffin and sectioned for hematoxylin and eosin (HE) staining, as previously described [36, 60]. In order to identify bacterial antigen, immunohistochemistry was performed on selected formalin fixed tissues as previously described using a rabbit polyclonal antibody for *Burkholderia* spp. exopolysaccharide [61]. Representative immunohistochemistry is depicted in S1 Fig. We define a MNGC as a large (> 20μm diameter), round to irregular cell with abundant clear to eosinophilic cytoplasm and having two or more eccentric reniform nuclei. N = 3 mice for pathological analyses for most time points.

### Spleen cell preparation

Splenocytes were prepared as previously described [62]. Briefly, spleens were excised from mice (N = 5 mice for most time points), weighed, and disaggregated in RPMI 1640 medium (Life Technology, Grand Island, NY) containing 25 mM HEPES, 2 mM glutamine (wash medium) to make the spleen extract. Aliquots of the spleen homogenate were saved for cytokine/chemokine determination and stored at -70° C. Samples were irradiated with approximately 2.1 kGy of gamma-radiation and confirmed sterile by testing 10% of the sample before use. CFU in non-irradiated aliquots of the homogenate were determined on sheep blood agar plates with undiluted extract or 10-fold dilutions in sterile GTB. Plates were incubated at 37° C for two days before counting CFU. Red blood cells in the spleen homogenate were lysed with ACK (Ammonium-Chloride-Potassium) Lysing Buffer (BioWhittaker, Walkersville, MD) after the extract was diluted with wash medium and cells pelleted by centrifugation at 1200 rpm for 10 min. Splenocytes were washed and suspended in complete medium [wash medium containing 10% heat-inactivated fetal calf serum (Life Technology), 1 mM sodium pyruvate, 0.1 mM non-essential amino acids, 100 U/ml of penicillin, 100 μg/ml streptomycin, and 50 μM 2-mercaptoethanol and cells counted.

### Cytokine/chemokine expression

Cytokines and chemokines in mouse sera and spleen homogenates (N = 5 for most time points) were measured by Luminex Mag Pix (Life Technology, Grand Island, NY) as per manufacturer directions. Spleen homogenates and sera from uninfected mice were used as normal, uninfected controls (N = 10 BALB/c; N = 4 C57BL/6). The levels (pg/ml) of the following 20 cytokines/chemokines were measured: FGFb, GM-CSF, IFN-γ, IL-1α, IL-1β, IL-2, IL-4, IL-5, IL-6, IL-10, IL-12 (p40/p70), IL-13, IL-17, IP-10, KC, MCP-1, MIG, MIP-1α, TNF-α, and VEGF. We did not report all the cytokines/chemokines because some did not show any change during the study.

### Splenocyte composition

Approximately 1x10^7^ splenocytes from each mouse were washed in FACS staining buffer (FSB) (1XPBS, 3% fetal calf serum, Life Technologies), and fixed in FSB containing 4% formaldehyde (Pierce, Rockford, IL) at 4° C. The cells were washed in FSB and then distributed into a microtiter plate (5x10^5^ cells/well), and nonspecific binding was inhibited by the addition of Fc Block (BD Biosciences, San Jose, CA). Cells were labeled with the following specific antibodies (BD Biosciences): CD4 T cells, CD4-PE/CD44-FITC; CD8 T cells, CD8-PE/CD44-FITC; B cells, B220-PE/CD86-FITC; monocytes/macrophages, CD11b-PE/CD44-FITC; NK cells, CD49b-PE/CD44-FITC; and granulocytes, Ly6G-PE/CD44-FITC. Corresponding isotype controls were used, and all were incubated for 60 min on ice. Cells were identified with a BD FACSCalibur using CellQuestPro software (BD Biosciences). Splenocytes from uninfected BALB/c mice were prepared as described above and used as controls.

### Antibody ELISAs

Immunoglobulin (Ig) class IgG titers in challenged mice were determined by an ELISA performed in 96-well, Immulon 2 HB, round-bottom plates (ThermoFisher). Irradiated *B*. *pseudomallei* K96243 cells (used as antigen) were diluted in 0.1 M carbonate buffer, (pH 9.5) to a concentration of 10 μg/ml. 50 μl of diluted irradiated cells were placed into wells. Plates were stored overnight at 4° C. The plates were washed with washing solution (1× PBS, 0.05% Tween 20), and incubated with 100 μl of blocking solution (1× PBS, 1% Casein) for 30 min at 37° C. Two-fold dilutions of mouse sera were made with antibody assay diluent (1X PBS, 0.25% casein) in triplicate, and plates were incubated for 1 h at 37° C. After the plates were washed, 50 μl of 1/5,000-diluted anti-IgG-horseradish peroxidase conjugate (obtained from Southern Biotechnology Associates, Inc. Birmingham, AL) was added to each well, and plates were incubated for 30 min at 37° C. After the plates were washed, 50 μl of a buffered hydrogen peroxide and 3,3′,5,5′-tetramethylbenzidine solution (Pierce, ThermoFisher) was added to each well, and plates were incubated for 20 min at 37° C. The reaction was stopped with 25 μl of 2 N sulfuric acid, and the amount of bound antibody was determined colorimetrically by reading at 450 nm with a reference filter (570 nm). The results are reported as the reciprocal of the highest dilution giving a mean OD of at least 0.1 (which was at least twice the background) ± 1 SD.

### Statistical analyses

Individual daily temperatures and weights were analyzed. The weights were analyzed by repeated measures ANOVA. For temperatures, the 5 day lagged, moving average was computed by taking the average of each daily measure and the measures obtained on the 4 preceding days. The definition is extended to study days preceding the fifth by including all days available. The motivation in using these running averages is to reduce the impact of biologically implausible fluctuations apparent in the individual temperature profiles. The resulting lagged averages were entered into a linear mixed effect repeated measures model for analysis. The model utilized a moving average correlation structure to accommodate the inherent correlation among the lagged averages. The analysis is implemented in SAS^®^ Proc Mixed. Bacterial burdens are depicted as CFU per gram of tissue. The geometric means of the calculated values are included as horizontal bars. Spleen weights, cytokines, and cell distributions were log transformed prior to analysis by Welch’s T-test. *P*-values are not adjusted for multiple comparisons.

## Results

### Mice challenged IP with *Burkholderia pseudomallei* K96243

#### The impact of IP bacterial challenge on body temperature and weight of mice

**Fig 1:**
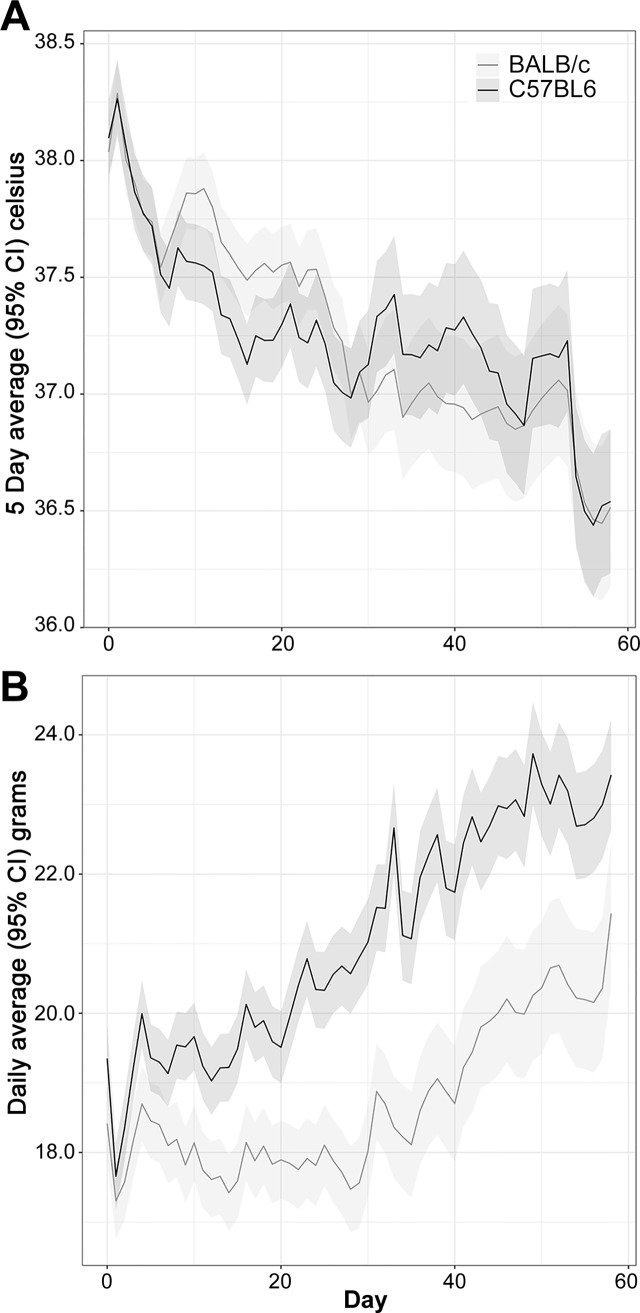
Analyses of daily recorded temperatures (A) and daily recorded weights (B) for mice challenged with *B*. *pseudomallei* K96243 delivered via the IP route.

Individual temperatures and body weights were recorded daily following IP challenge with *B*. *pseudomallei* K96243 (S1 Table). The BALB/c mice had a greater body temperature compared to C57BL/6 mice starting at day 10 and continuing through day 20, but differences were not statistically significant thereafter (Fig 1A). Notably, BALB/c mice showed a 0.3°C increase in body temperature when compared to the C57BL/6 mice at these early time points, an observation which closely mirrors that obtained in mice exposed to aerosolized bacteria (discussed later). The time by strain interaction was statistically significant (*P* < 0.01), confirming that an overall difference in the temperature profiles between the two strains existed.

The mouse weights for BALB/c and C57BL/6 mice were statistically distinguishable starting at day 5 and at every time point thereafter (Fig 1B). At day 0 the mouse strains differed by 0.5 grams, an amount which was not statistically significant (*P* = 0.71). The C57BL/6 mice exhibited a greater average weight gain relative to the BALB/c mice, leading to a statistically significant time by strain interaction (*P* < 0.01).

#### Bacterial burden observed in mice receiving an IP injection of bacteria

**Fig 2:**
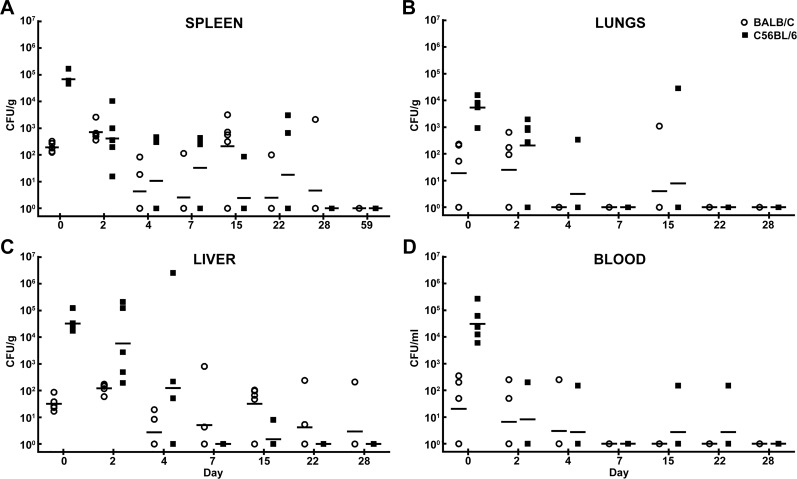
Bacterial burden determined in mice challenged with *B*. *pseudomallei* K96243 delivered via the IP route. CFU/g for spleen **(A)**, lungs **(B)**, liver **(C)** and CFU/ml for blood **(D)** are depicted. The geometric mean for each group is indicated by a horizontal bar. BALB/C mice are depicted with open circles and C57BL/6 mice are depicted with filled squares. N = 5 at each time point.

Fig 2 illustrates the recovered CFU/gram of organ or CFU/ml of blood following IP challenge. Similar median lethal dose equivalents were administered to either the BALB/c (approximately 3.0x10^4^ CFU or 0.49 LD_50_ equivalent) or C57BL/6 mice (approximately 9.2x10^5^ CFU or 0.42 LD_50_ equivalent); to account for the inherent differences in susceptibility to infection that have been well documented [34, 35, 63]. The dissemination patterns observed in either BALB/c or C57BL/6 mice were similar (Fig 2). Of interest is the rapid hematogenous spread of bacteria throughout the animal. Bacteria were identified in all organs and blood in most mice on day 0 (within approximately 4–6 hours post injection). As demonstrated in previous reports, spleen weight can be indicative of aspects of both bacterial replication and host immune response [32, 33]. Thus, we compared the weights of the spleens obtained in BALB/c mice and C57BL/6 mice. As shown in S2 Fig, in the IP challenge experiment, spleen weights and associated weight increases were statistically indistinguishable, with the exception being day 4 post-infection. Throughout the course of the study, some of the mice were euthanized in accordance with early endpoint criteria or succumbed to infection (19 of 80 BALB/c mice and 13 of 80 C57BL/6 mice).

#### Histopathology observed in mice receiving an IP injection of bacteria

**Fig 3:**
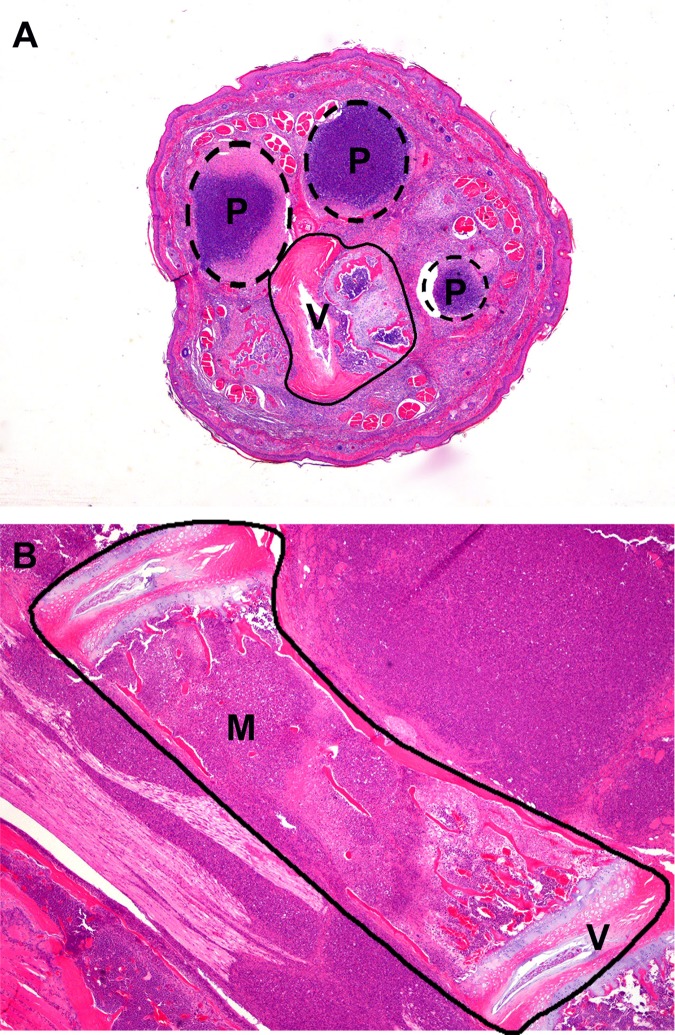
Histopathology observed in mice with rear leg clinical signs assocaited with IP challenge with *B*. *pseudomallei* K96243. **(A)** C57BL/6 euthanized on day 22 post-infection showing clinical signs in the hind-end and tail. Tail, transverse section: Multiple pyogranulomas partially effacing vertebral body and associated soft tissues. H&E, 20X. **(B)** BALB/c mouse euthanized on day 25 post-infection with rear-leg paralysis and labored breathing. Lumbar spine, longitudinal section: Pyogranulomatous inflammation partially effacing vertebral body and associated soft tissues. P = pyogranuloma; M = marrow cavity; V = vertebral body; H&E, 40X.

Consistent with previous studies involving IP infection of *B*. *pseudomallei* in mice [36], the most striking lesions attributed to *B*. *pseudomallei* infection in 9/36 C57BL/6 and 17/36 BALB/c mice were focally extensive areas of pyogranulomatous inflammation in the rear legs, tail, and spine (Fig 3A). It is unclear why there is an apparent predisposition for the caudal half of the body (rear legs, tail, and caudal vertebral column); however, it may be related to the route of lymphatic drainage from the IP challenge site. These lesions in mice were first observed within a week of each other post exposure in both mouse strains, beginning in BALB/c mice at day 15 and C57BL/6 mice at day 22. However, lesions in BALB/c mice were seen with more frequency and increased severity. Lesions were composed of large aggregates of viable and degenerate neutrophils and necrotic debris surrounded by low numbers of epithelioid macrophages. The areas of pyogranulomatous inflammation (Fig 3B) were widespread and indiscriminant about the tissue types affected, including skeletal muscle, peripheral nerves, bone, cartilage, adipose tissue, and fibrous connective tissue. This situation made determination of the temporal pathogenesis of these lesions difficult; however, it is likely that these lesions represent persistent niduses of inflammation incited by hematogenous or lymphatic bacterial spread earlier in the course of disease. Human case reports have documented *B*. *pseudomallei* infection in muscle, bones, and joints [64, 65]. Other less common but significant sites of pyogranulomatous or suppurative inflammation included the cerebrum, cerebellum, brainstem, liver, and spleen. Lesions in these tissues illustrate the widely dispersed sites of inflammation resulting from hematogenous spread of *B*. *pseudomallei* following IP challenge.

**Fig 4:**
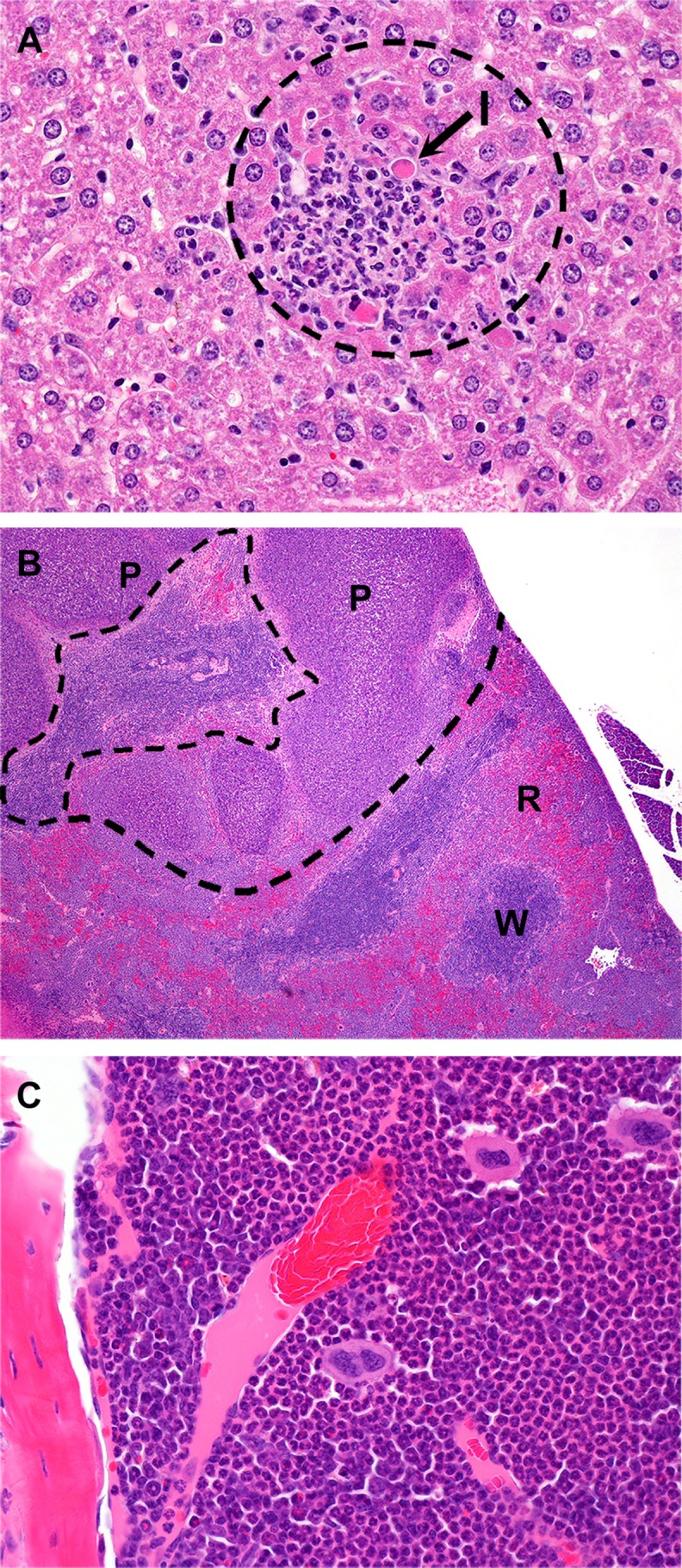
Histopathology observed in mice following IP challenge with *B*. *pseudomallei* K96243. **(A)** C57BL/6 mouse euthanized on day 2 post-infection Liver: Random foci of neutrophilic inflammation with individual hepatocyte necrosis/apoptosis (arrow). I = inflammation; H&E, 400X. **(B)** BALB/c mouse euthanized on day 20 post-infection with rear-leg paralysis. Spleen: Multiple pyogranulomas effacing red and white pulp; H&E, 40X. **(C)** C57BL/6 mouse euthanized on day 22 post-infection. Femoral bone marrow: Myeloid hyperplasia with predominance of neutrophils. H&E, 400X. P = pyogranuloma; R = red pulp; W = white pulp;

Inflammation in the liver, which was the earliest detectable lesion in both mouse strains, began acutely as neutrophilic infiltration of hepatic sinusoids and areas of individual hepatocyte necrosis (Fig 4A) and was present in 6/6 C57BL/6 mice and 5/6 BALB/c mice between days 0 and 4. This progressed to mixed neutrophilic and histiocytic infiltration, and in a few mice, to suppurative or pyogranulomatous hepatitis in 18/30 C57BL/6 and 13/30 BALB/c mice from day 7 until day 59. Immunohistochemistry demonstrated large amounts of *B*. *pseudomallei* exopolysaccharide antigen in these areas of pyogranulomatous inflammation (S1 Fig).

Neutrophilic inflammation was only seen acutely in the spleen of 1/6 BALB/c mice and was not seen acutely in C57BL/6 mice, although the identification of these lesions was often obfuscated by the striking extramedullary hematopoiesis (EMH) seen in these mice. The development of pyogranulomas and abscesses within the spleen (Fig 4B) was only seen in 4/36 BALB/c mice but was not seen in any of the C57BL/6 mice. While EMH in the splenic red pulp and sinusoids of the liver are very commonly seen in normal mice in response to a variety of antigens [66], the degree of EMH in these mice was significantly greater than what is typically encountered and affected 28/36 C57BL/6 and 19/36 BALB/c mice between days 4 and 59. This is consistent with a physiologic response to increased tissue demand for leukocytes secondary to bacterial infections that elicit intense inflammatory reactions. Given the large areas of pyogranulomatous inflammation seen in these mice, this exuberant EMH is most likely related to infection with *B*. *pseudomallei*. For the same reason, many of these mice had significant myeloid hyperplasia in the bone marrow (Fig 4C), predominantly of the neutrophil lineage. In some cases, the myeloid hyperplasia was so intense that it extended outside of the marrow cavity of the bones and into adjacent tissues. In the case of the vertebral column, this excessive hyperplasia occasionally resulted in compression and/or disruption of the spinal cord and peripheral nerve ganglia. This may partially explain why some mice, despite a lack of significant pyogranulomatous inflammation in the spine or rear limbs, still exhibited neurologic clinical signs (i.e. paralysis, ataxia). Interstitial neutrophilic inflammation was seen in the lung of 13/36 C57BL/6 and 17/36 BALB/c mice. The pathogenesis of this inflammation in the lung is not clear. There is little histologic evidence that the lung is a primary site of *B*. *pseudomallei* infection in these mice by IP challenge, as none of the C57BL/6 mice only 3/36 BALB/c developed suppurative or pyogranulomatous pneumonia at any time during the study. The increased neutrophils observed in some of the lung samples could be confined to the capillaries in the interstitium and represent the relative increase in numbers of circulating neutrophils in the blood as a response to inflammation elsewhere in the body.

#### Immunological response observed in mice receiving *B*. *pseudomallei* K96243 by IP injection

**Fig 5:**
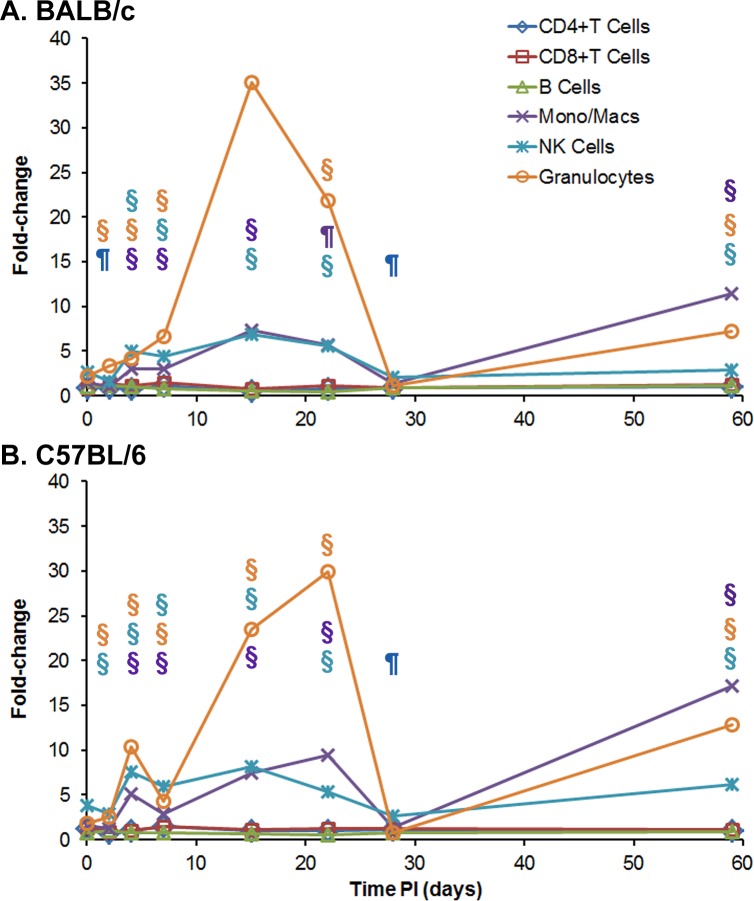
Cellular changes in spleens occurring in BALB/c and C57BL/6 mice after IP infection with *B*. *pseudomallei* K96243. Spleen homogenates were prepared from **(A)** BALB/c and **(B)** C57BL/6 mice over time after infection, and the percent of each cell type examined was determined. For each mouse strain, N = 5 at each time point. The fold-change for each cell type was determined by dividing the percent of the cell type at each time point (S2 Table) by the percent of the cell type present in normal, naïve mice, where N = 10 for BALB/c and N = 4 for C57BL/6 mice. Significant changes in % cell type (S2 Table) are shown above the inflammatory cells (macrophages/monocytes, NK cells, and granulocytes) only because they showed the most predominate changes throughout the study. Significant levels compared to the naïve, control mice: ¶ *P* < 0.01; § *P* ≤ 0.001.

We examined the cellular immune response in spleens of the infected mice after IP infection. We used the same spleens that were utilized in the previous analyses (CFU burdens and weight) to examine the changes in the cellular composition of the spleens after infection over time. We also analyzed cytokine/chemokine expression in serum and spleen extracts from the same mice was assessed. All days described below represent days post exposure to *B*. *pseudomallei*. The histopathology described above of tissue/organs noted the large increase in neutrophils after IP infection in BALB/c mice. We used flow-cytometry to better identify and quantitate the different types of cells that infiltrate the infected spleens after IP infection (S2 Table and Fig 5). We compared the cellular composition of the infected *B*. *pseudomallei* K96243 mouse spleens to the cellular composition of spleens from normal, naïve mice (S2 Table, Fig 5A). Within 4–6 hours post exposure (day 0), there was a slight increase in monocytes/macrophages (CD11b+/CD44) and granulocytes (Ly6G+/CD44) approximately1.5-fold-2.1-fold, respectively followed by NK cells (CD49b+/CD44) (2.7-fold, *P* ≤ 0.001). We detected a slight but significant increase in monocytes, macrophages, NK cells, and granulocytes (3.0-fold, *P* ≤ 0.001; 5.0-fold, *P* ≤ 0.001; and 4.2-fold, *P* < 0.001, respectively) at day 4. Between 7 to 15 days, we saw a significant increase in the inflammatory, monocytes/macrophages (7.3-fold, *P* ≤ 0.001), NK cells (6.9-fold, *P* < 0.01) and granulocytes which peaked at this time (35.1 fold, *P* ≤ 0.001)in spleens of BALB/c mice where they then decreased by day 22, but remained statistically higher than those observed in uninfected control mice (*P* < 0.01, *P* ≤ 0.001, and *P* ≤ 0.001, respectively). On day 28 the amount of the three inflammatory cell types dropped close to levels seen at day 0 in the spleens from BALB/c mice. This was followed by a significant increase in the monocytes/macrophages (*P* ≤ 0.001), NK cells (*P*≤0.001), and percentage of granulocytes (*P* < 0.01), until the end of the study at day 59. During the same period of the study, the three other cell types that we examined, CD4+ and CD8+ T cells, and B cells, we detected only a slight but modest overall increase in CD8+ T cells at days 7 and 59 (1.5-fold, *P* ≤ 0.001; and 1.3-fold, *P* < 0.05), respectively)(S2 Table and Fig 5A).

In C57BL/6 mice, we detected a slightly different pattern in the increase in inflammatory granulocytes, monocytes/macrophages, and NK cells in the early part of the infection (S2 Table and Fig 5B). On day 4, we detected a significant increase in the inflammatory cell types (10.4-fold, *P* ≤ 0.001; 5.1-fold, *P* ≤ 0.001; and 7.5-fold, *P* ≤ 0.001, respectively) before they decreased at day 7. Then at day 15, we saw an increase in the amount of granulocytes (23.5-fold, *P* ≤ 0.001) present in the infected spleens, with a further increase and peak detected at day 22 (29.9-fold, *P* ≤ 0.001). We detected a lower but significant increase in monocytes/macrophages from 7.4-fold (*P* ≤ 0.001) to 9.4-fold (*P* ≤ 0.001) at days 15 and 22, respectively. For the same two days post exposure, we saw a significant increase in NK cells of 8.21-fold (*P* ≤ 0.001) and 5.3-fold (*P* ≤ 0.001), respectively. Similar to what we observed in BALB/c spleens at day 28, we detected lower amounts of granulocytes, monocytes/macrophages, and NK cells (0.8-fold, 1.3 fold, and 2.7-fold, respectively) compared to that found in the normal, naïve C57BL/6 spleens. We then detected a significant increase in granulocytes (12.8-fold, *P*≤0.001), monocytes/macrophages (17.2-fold, *P* ≤ 0.001), and NK cells (6.1-fold, *P* ≤ 0.001) after 59 days. Although we observed modest but significant changes in the number of CD4+ and CD8+ T cells and B cells over the same period of the study (S2 Table and Fig 5B), they were not as large as seen in the inflammatory cells.

Overall, the pattern of increase in the inflammatory cells (primarily granulocytes, followed by monocytes/macrophages and NK cells) in spleens from infected BALB/c mice was similar to that seen in infected spleens from C57BL/6 mice, except for the early influx in these cells at day 4 that we observed in infected spleens from C57BL/6 mice (Fig 5B). We observed that granulocytes reached a peak level on day 15 in BALB/c mice but the granulocyte peak was reached on day 22 in C57BL/6 mice. There was a similar substantial decrease in the three inflammatory cell types in spleens from both species of mice between 22 to 28 days before we saw an increase of these cell types was observed by the end of the study on day 59.

We also examined the change in cytokine/chemokine levels in sera and spleen extracts in both types of mice after IP infection. The amounts of 15 out of 20 cytokines/chemokines (reported as geometric means with geometric standard error of the means) that we detected in sera from infected BALB/c mice is shown in S3 Table. We did not report all 20 cytokines/chemokines that were measured because some did not show any changes (compared to the naïve, uninfected control mice) during the study. We saw immediate [day 0, 4–6 hours post exposure] changes in IL-1α [196.2 (1.16) pg/ml, *P* ≤ 0.001], IL-5 [36.7 (1.21) pg/ml, *P* < 0.05], and KC [2668 (1.15) pg/ml, *P* ≤ 0.001]. After 2 days, we detected a significant increase in sera of IFN-γ [168.3 (1.11) pg/ml, *P* ≤ 0.001], IL-1β [113.9 (1.12) pg/ml, *P* ≤ 0.001], FGFb [288 (1.05) pg/ml, *P* < 0.05], IP10 [93.3 (1.16) pg/ml, *P* ≤ 0.001], MCP-1 [37.2 (1.16) pg/ml, *P* < 0.05], and MIG [4009 (1.11) pg/ml, *P* ≤ 0.001]. These increases can be seen more clearly in S4A Fig, which shows the fold-changes in the cytokines/chemokines in serum. After 4 days, we saw little change in the levels of the cytokines/chemokines in sera until day 59 when we saw small increases in TNFα (2.39-fold, *P* < 0.05), IL-1β (3.76-fold, *P* < 0.05), IL-2 (2.81-fold, *P* ≤ 0.001), and IL-10 (2.88-fold, *P* ≤ 0.001).

In sera of C57BL/6 mice (S3 Table), we saw immediate (day 0, 4–6 h post exposure) increases over naïve controls of IL-1α [235.3(1.15) pg/ml, *P* ≤ 0.001], IL-5 [96.5 (1.19) pg/ml, *P* < 0.01], KC [3848 (1.08) pg/ml, *P* ≤ 0.001], MCP-1 [247.5 (1.29) pg/ml, *P* ≤ 0.001], and MIG [439.7 (1.33) pg/ml, *P* ≤ 0.001] (S3 Table). After day 2 we detected significant increases in IFN-γ [97.6 (1.11) pg/ml, *P <* 0.01], IL-1α [85.3 (1.33) pg/ml, *P <* 0.05], IL-5 [37.2 (1.20) pg/ml, *P* < 0.05], FGFb [516.9 (1.05) pg/ml, *P* < 0.05], IP-10 [78.0 (1.43) pg/ml, *P* < 0.05], KC [424.4 (1.68) pg/ml, *P* < 0.05], MCP-1 [118.0 (1.54) pg/ml, *P* < 0.05], and MIG [2107 (1.13) pg/ml, *P* ≤ 0.001]. At 4 days post exposure, we saw only increases in FGFb [464.2 (1.14) pg/ml, *P* < 0.05] and MIG [491.8 (1.27) pg/ml, *P* ≤ 0.001]. The early fold-changes in sera from C57BL/6 mice can be seen in S4B Fig when compared to those from BALB/c mice, and S3 Table. For MIG at 0, 2, and 4 days, there were increases of 54.3-fold (*P* ≤ 0.001), 235-fold (*P* ≤ 0.001), and 54.0-fold (*P* ≤ 0.001), respectively. For the chemokine KC at day 0 in C57BL/6 mice we saw there was an immediate 52.7-fold increase (*P* ≤ 0.001), which was high compared to the other cytokines/chemokines except for MIG (S3 Fig). Between days 7 to 28 levels of IFN-γ (*P* < 0.05), IL-1α (*P* < 0.01), and MIG (*P* ≤ 0.001) were significantly elevated compared to most of the other cytokines/chemokines. Finally, by day 59, we detected significant levels of TNF-α [44.8 (1.11) pg/ml (*P* < 0.01)], IL-2 [19.0 (1.03) pg/ml, *P* ≤ 0.001)], IL-10 [147.9 (1.07) pg/ml, *P* ≤ 0.001)], KC [225.4 (1.38) pg/ml, *P* < 0.05)], and MIG [77.3 (1.15) pg/ml, *P* ≤ 0.001)]. Generally, at the early time points, we saw a few more elevated cytokines/chemokines in the sera of BALB/c mice post exposure than in sera of C57BL/6, but there were slightly higher levels of inflammatory cytokines/chemokines after the initial peak seen post exposure in sera from C57BL/6 mice.

**Fig 6:**
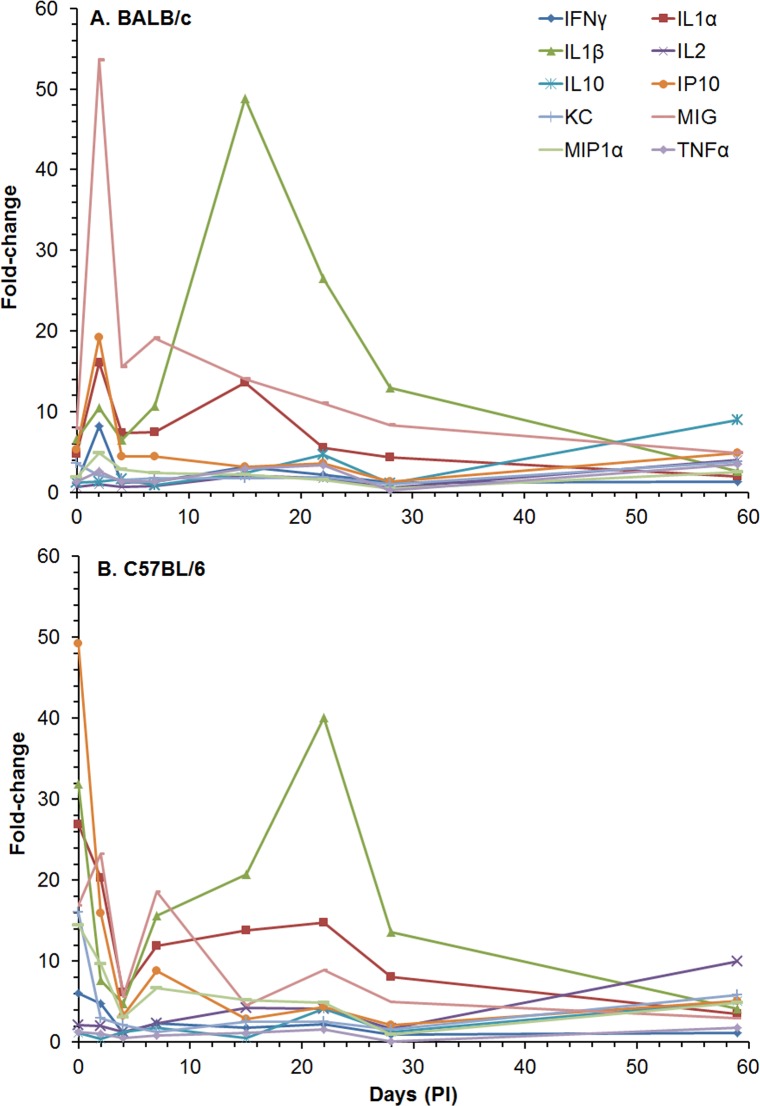
Changes in the amount of cytokines/chemokines in spleen extracts from BALB/c and C57BL/6 mice after IP infection with *B*. *pseudomallei* K96243. The amount of cytokines/chemokines present in spleen extracts (S4 Table) was determined. Only fold-changes in ten of the cytokines/chemokines are shown for **(A)** BALB/c and **(B)** C57BL/6 because they showed the most changes from normal levels after infection or were known to be important for host immunity, such as TNF-α. For each time point, N = 5 for BALB/c and C57BL/6 mice. Fold-changes in cytokines/chemokines was determined by dividing the amount (pg/ml) present in the spleen extract by the amount present in normal, naïve mice, where N = 10 for BALB/c and N = 4 for C57BL/6 mice.

We also examined the amount of cytokines/chemokines in spleen extracts post-infection from the same set of mice that we analyzed above (S4 Table and Fig 6). In spleen extracts from BALB/c mice at 0 day (4–6 h after exposure), we detected a significant increase in levels of a majority of the cytokines/chemokines compared to naïve mice: IL-1α [356.8 (1.09) pg/ml, *P* ≤ 0.001], IL-1β [256.8 (1.09) pg/ml, *P* ≤ 0.001], IL-12 [63.8 (1.03) pg/ml, *P* ≤ 0.001], FGFb [2908 (1.13) pg/ml, *P* ≤ 0.001], IP-10 [103.8 (1.08) pg/ml, *P* ≤ 0.001], KC [1237 (1.21) pg/ml, *P* < 0.01], MCP-1 [41.2 (1.15) pg/ml, *P* < 0.01], MIG [1122 (1.10) pg/ml, *P* ≤ 0.001], MIP-1α [108.9 (1.06) pg/ml, *P* ≤ 0.001], and VEGF [58.8 (1.06) pg/ml, *P* ≤ 0.001]. At day 2, we detected a significant increase in the same cytokines/chemokines as day 0 with the addition of IFN-γ [171.4 (1.16) pg/ml, *P* ≤ 0.001], TNF-α [33.2 (1.06) pg/ml, *P <* 0.001], IL-5 [42.3 (1.14) pg/ml, *P* < 0.05], IL-6 [48.1 (1.08) pg/ml, *P* ≤ 0.001], and IL-10 [35.2 (1.07) pg/ml, *P* ≤ 0.001]. At days 4 and 7, we saw fewer increases in the cytokines/chemokines levels, but at day 15, we saw the greatest increase in the amount and number of cytokines/chemokines expressed (S4 Table and Fig 6A). The peak of activity began decreasing after day 15 and further decreased by day 22 before we detected a slight but significant increase in the amount and number of cytokines/chemokines expressed in spleen extracts on day 59.

When we examined the amount of cytokines/chemokines present in spleen extracts from C57BL/6 mice, we saw at least three differences over the course of the study compared to that in BALB/c spleen extracts (S4 Table and Fig 6B). First, in most cases there was a greater immediate increase in relative amounts of examined cytokines/chemokines that we examined in spleen extracts from C57BL/6 that we detected on day 0 (4–6 h after exposure), that were higher than found in spleen extracts from BALB/c mice. Most notable were IP-10 [1221 (1.25) pg/ml, *P*≤0.001], IL-1β[672.7 (1.10) pg/ml, *P*≤0.001], IL-1α [1237 (1.25) pg/ml. *P*≤0.001], MIG [3653 (1.21) pg/ml, *P*≤0.001], KC [3320 (1.12) pg/ml, *P*≤0.001], MIP-1α [382.4 (1.25) pg/ml, *P*≤0.001], and IFN-γ [100.3 (1.35) pg/ml, *P* < 0.01]. Second, we did not see a peak in the change in the level of the cytokines/chemokines (mentioned above) on day 2, as we saw in spleen extracts from BALB/c mice, but there was a modest peak at day 7 (Fig 6B). The third difference was that the peak change in IL-1β [846.5 (1.67) pg/ml, *P* < 0.01] levels, which is an inflammatory cytokine, occurred at day 22 in spleen extracts from C57BL/6 infected mice, rather than at day 15 in spleen extracts from infected BALB/c mice. However, there was a distinct decrease in the change in the levels of cytokines/chemokines at day 28 seen in spleen extracts from both strains of mice. We also detected a similar increase in many of the cytokines/chemokines on day 59 in spleen extracts from both mice (S4 Table). Hence, we saw an increase in the inflammatory cytokines IL-1α and IL-1β and also MIG was increased in the early part of the infection in extracts from both strains of mice. As we saw in the change in the influx of inflammatory cells in the spleen (Fig 5), there appeared to be a distinct change in cytokines/chemokines levels between 22 and 28 days post exposure in spleen extracts that may suggest that there was a transition from an early or acute phase of infection to a late or chronic phase of infection (Fig 6).

### Mice exposed to aerosolized *Burkholderia pseudomallei* K96243

#### The impacts of exposure to aerosolized *B*. *pseudomallei* on body temperature and weights of mice

**Fig 7:**
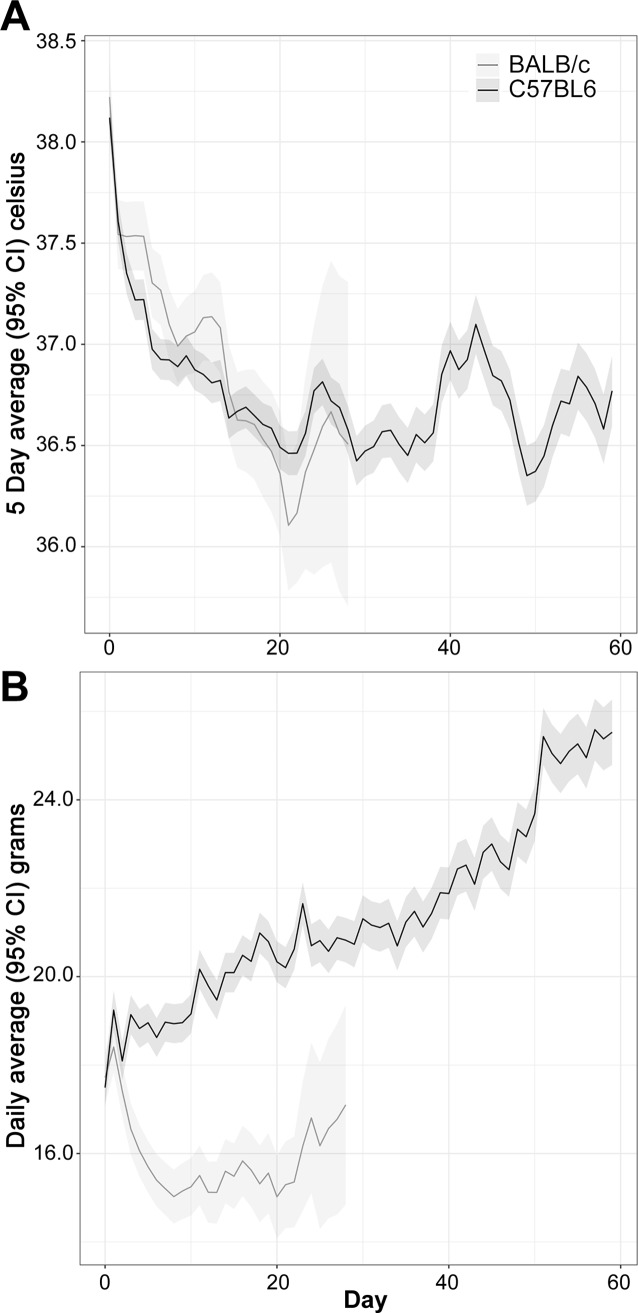
Analyses of daily recorded temperatures (A) and daily recorded weights (B) for mice exposed to aerosolized *B*. *pseudomallei* K96243.

Individual weights and temperatures were recorded daily (S5 Table). When mice were exposed to aerosolized bacteria differences in temperature between BALB/c and C57BL/6 mice were observed. Statistically significant differences between the BALB/c and C57BL/6 mouse body temperatures were found for the average of day 1 to day 5 (*P* < 0.01), with the BALB/c strain having a temperature 0.33°C greater than that of the C57BL/6 mice (Fig 7A), however differences between the BALB/c and C57BL/6 strains were not statistically significant at later time points. The strain by time interaction was statistically significant (*P* < 0.01), which is attributable to the separation at early time points diminishing as the study continued. Statistically significant differences in body weight between the mouse strains were observed by day 5 and at all subsequent time points (Fig 7B). The strain by time interaction was statistically significant (*P* < 0.01). Absolute differences in mean body weight between strains continued to increase with time, reaching 4.5 grams by day 15.

#### Bacterial burden observed in mice exposed to aerosolized *Burkholderia pseudomallei*

**Fig 8:**
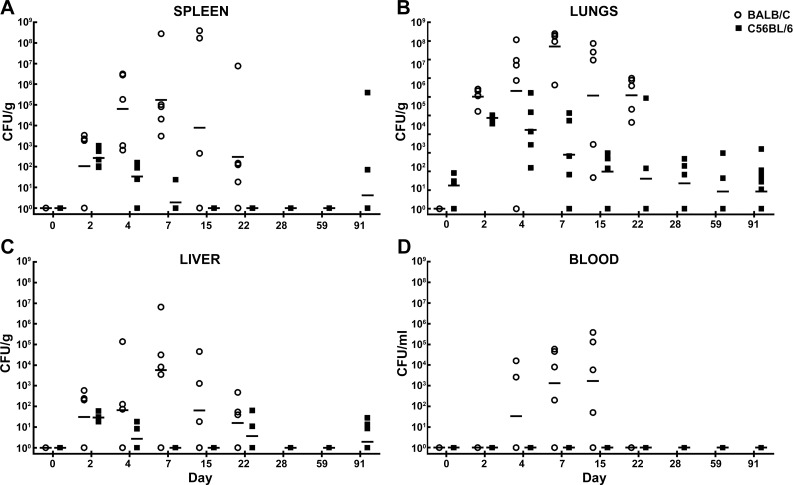
Bacterial burden determined in mice exposed to aerosolized *B*. *pseudomallei* K96243. CFU/g for spleen **(A)**, lungs **(B)**, liver **(C)** and CFU/ml for blood **(D)** are depicted. The geometric mean for each group is indicated. BALB/C mice are depicted with open circles and C57BL/6 mice are depicted with filled squares. N = 5 at each time point through day 22 for both mouse strains. The surviving BALB/c mice after day 22 were used to perform histopathological analyses. N = 12 for surviving C57BL/6 mice on day 91.

Unlike the dissemination patterns observed in mice exposed to *B*. *pseudomallei* via IP injection, the mice that were exposed to aerosolized *B*. *pseudomallei* had fairly distinct dissemination patterns that differed between BALB/c and C57BL/6 mice. Some of these differences are likely partially related to the different LD_50_ equivalents delivered via each route of exposure. Both strains of mice received low doses of aerosolized bacteria; the BALB/c were exposed to approximately 5 CFU (0.2 LD_50_ equivalent) and the C57BL/6 mice were exposed to approximately 18 CFU (0.05LD_50_ equivalent). BALB/c mice were all euthanized or had succumbed to infection by day 28, whereas the C57BL/6 mice survived longer (despite receiving a more than three-fold greater number of CFU) and serial samples were collected up to day 91. The dissemination patterns for the BALB/c mice were similar in all organs sampled (Fig 8). While variation existed between animals, the average bacterial burden seemed to peak at day 7 post-exposure and then continue to drop through day 22. The bacterial burden in the lungs was the most pronounced (Fig 8B), followed by the spleens (Fig 8A) and then finally liver samples (Fig 8C). The liver samples indicated that bacterial burden in this organ was approximately 10% of what can be observed in the spleens of the same mice. Similar observations have been reported by Massey et al. [46]. Some of the BALB/c mice became bacteremic between day 2 and day 4 (Fig 8D).

The dissemination patterns of the C57BL/6 mice were markedly different compared to BALB/c (Fig 8). At time points examined, none of the mice showed signs of bacteremia (limit of detection 100 CFU/1 ml of blood) (Fig 8D). The C57BL/6 mice were seemingly better able to control the ensuing infection, and the bacterial burdens did not reach the magnitude observed in BALB/c mice and appeared to peak 2–4 days post-exposure to aerosolized bacteria. These dissemination data collected from the spleens (Fig 8A), lungs (Fig 8B), and livers (Fig 8C) of the C57BL/6 mice suggested a predisposition towards a potentially chronic infection as has been previously reported [27, 34]. The spleen samples (Fig 8A), for example, were negative (limit of detection 10 CFU/ml of spleen homogenate) after day 7 post-exposure. Twelve mice were euthanized and sampled on day 91 of which 2 of 12 mice were culture positive in the spleen sample (Fig 8A), 3 of 12 mice were culture positive in the liver sample (Fig 8C), and 6 of 12 mice maintained low levels of infection in lung tissue (Fig 8B). These data indicated that at least a subset of C57BL/6 mice retained a low level of bacteria 91 days post exposure to a low dose of aerosolized bacteria.

Spleen weight was also analyzed and compared between the two mouse strains, and significant differences were noted on days 15 and 22 post-infection (*P* = 0.03 and 0.0007, respectively), indicating that the BALB/c spleens were larger which in this experiment seemed to be associated with bacterial replication and possibly with the influx of inflammatory cells (S3 Fig). Throughout the course of the study, mice were euthanized in accordance with early endpoint criteria or succumbed to the infection (30 of 80 BALB/c mice and 1 of 80 C57BL/6 mice).

#### Histopathology observed in mice exposed to aerosolized bacteria

**Fig 9:**
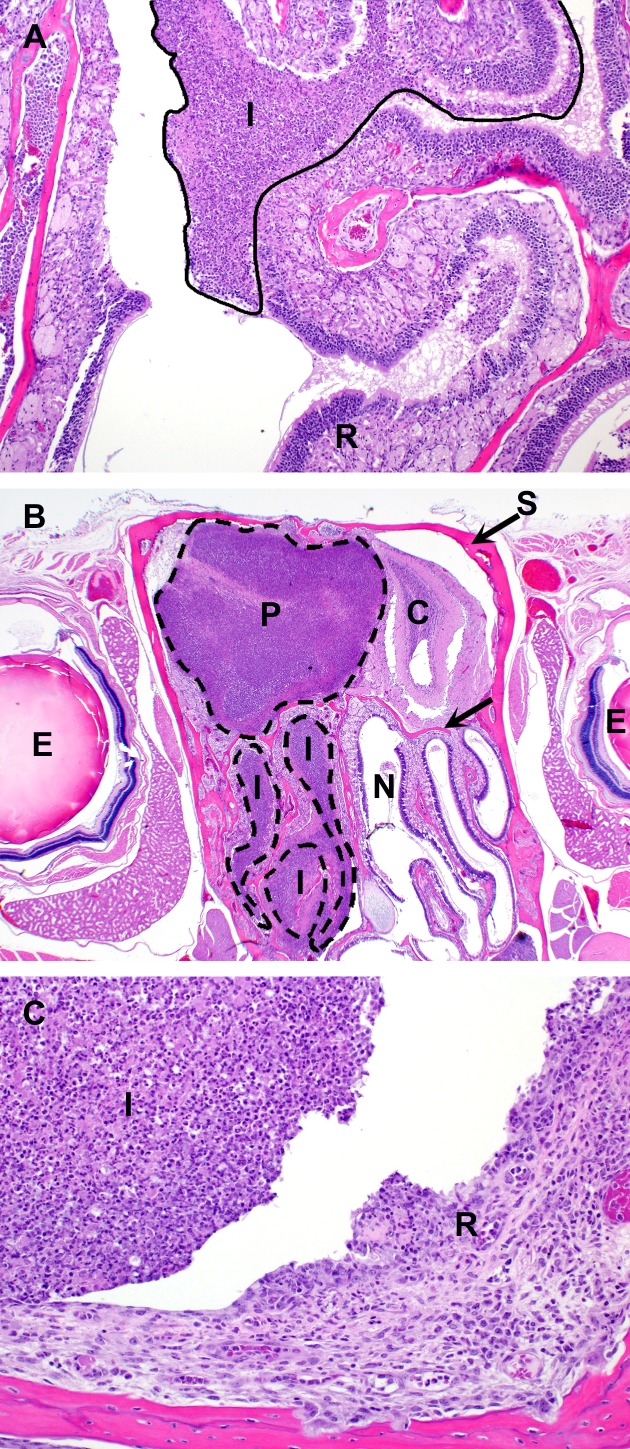
Cranial histopathology observed in mice following exposure to aerosolized *B*. *pseudomallei* K96243. **(A)** BALB/c mouse euthanized on day 4 post-infection. Nasal cavity: Epithelial and subepithelial suppurative inflammation and necrosis. R = respiratory epithelium; I = inflammation; H&E, 100X. (**B)** BALB/c mouse euthanized on day 10 when early endpoint-euthanasia criteria were met. Nasal cavity and calvarium: Suppurative inflammation arising in the nasal cavity (N) and extending through the cribriform plate (arrow) into the olfactory bulb and cerebrum (C). E = eye; S = skull; P = pyogranuloma; I = inflammation; H&E 20X. **(C)** BALB/c mouse euthanized on day 7 post-infection with clinical signs indicative of an inner ear-infection Middle ear: Suppurative inflammation and necrosis of epithelium (suppurative otitis media). I = inflammation; R = ulcerated respiratory epithelium; H&E 200X.

In contrast to the IP challenge model, mice in the aerosol challenge model most consistently developed acute lesions in the nasal cavity and lung and chronic lesions in the lung and spleen. Acute lesions in the nasal cavity were noted in 13/28 C57BL/6 mice as early as day 2 and in 9/29 BALB/c mice as early as day 4 and characterized by intense neutrophilic inflammation which filled nasal sinuses (Fig 9A) and occasionally caused necrosis of the respiratory/olfactory epithelium and underlying subepithelial connective tissue. These lesions were generally confined to the posterior segment of the nasal cavity and often directly abutted the cribriform plate. In 2 of the BALB/c mice, the inflammation continued along olfactory nerve tracts, penetrating the cribriform plate and involving the meninges and neuropil of the olfactory bulbs and rostral cerebrum (Fig 9B). This was accompanied by a marked to severe neutrophilic exudate in the middle ear (Fig 9C), with necrosis of the respiratory epithelium lining the middle ear in 8/28 C57BL/6 mice and 9/29 BALB/c mice. It is surmised that in these cases the inflammation in the middle ear originated in the nasal cavity and extended along the eustachian tubes into the middle ear. This phase of infection appears to have remained active beyond the acute post-challenge timeframe in at least some of the mice, as evidenced by the persistence of neutrophilic inflammation and the lack of a progression to a more chronic inflammatory cell population in animals as late as 91 days post-infection. This nidus of infection and inflammation may be a potential source of dissemination or reinfection for these mice at later time points.

**Fig 10:**
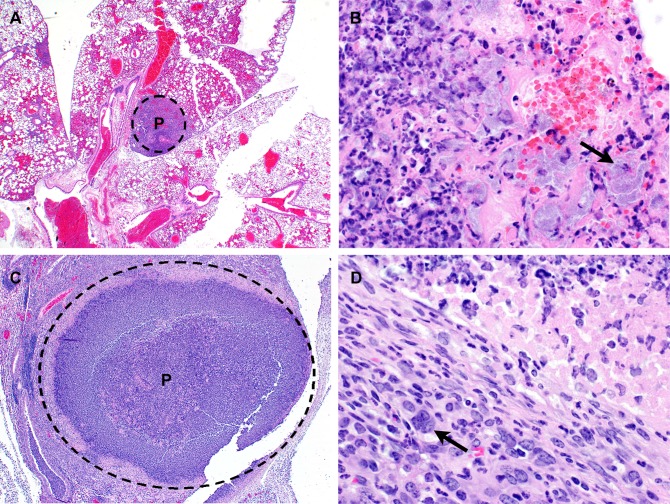
Lung histopathology observed in mice following exposure to aerosolized *B*. *pseudomallei* K96243. **(A)** C57BL/6 mouse euthanized on day 2 post-infection. Lung: Multifocal random (embolic) suppurative pneumonia. H&E 20X. **(B)** BALB/c mouse euthanized on day 4 post-infection. Lung: Suppurative inflammation and alveolar necrosis with numerous short bacilli (arrow). P = pyogranuloma; H&E 600X. **(C)** BALB/c mouse euthanized on day 7 post-infection. Lung: Focally extensive pyogranuloma. P = pyogranuloma; H&E 40X. **(D)** Lung: Periphery of pyogranuloma with MNGC macrophage (arrow) formation. H&E 600X.

It was observed that 16/28 C57BL/6 mice and 24/29 BALB/c mice had lung lesions attributed to *B*. *pseudomallei* infection. Not surprisingly, lesions in the lungs of the aerosolized mice from both strains occurred much more acutely than in mice challenged via the IP route. The earliest lesions consisted of multiple randomly arranged neutrophilic/suppurative foci with variable amounts of pneumocyte and septal necrosis. These foci were often associated with small and medium pulmonary vessels in an apparent embolic pattern (Fig 10A and 10B); this pattern of inflammation in the lung is usually associated with infectious agents that arrive via a hematogenous (embolic) route. This is unexpected, as one would expect the aerosolized *B*. *pseudomallei* to arrive as inhaled particles and establish lesions more consistent with bronchopneumonia. There are several possible explanations for this pattern of inflammation. The first is that following exposure to aerosolized *B*. *pseudomallei*, the bacteria quickly enter the circulation via alveolar septa and establish a bacteremia, with subsequent embolic spread to multiple sites throughout the lung and more distant organs. A second possibility is that following aerosol exposure, the bacteria are quickly phagocytized by resident alveolar macrophages, which then traffic the bacteria to these perivascular sites where they incite an intense neutrophilic reaction. A third possibility is that following whole body aerosol exposure, a bacteremia was established following ingestion of bacteria during grooming, which then spread embolically to the lungs. Given the short amount of time in which these lesions are established, the alveolar macrophage hypothesis seems more plausible, particularly since *B*. *pseudomallei* is hypothesized to evade the immune response by its intrahistiocytic localization [67]. In the BALB/c mice, by day 7 the inflammation progressed to histiocytic or pyogranulomatous inflammation (Fig 10C and 10D), with higher numbers of histiocytes and epithelioid macrophages. In some cases, these areas of granulomatous inflammation developed into well-organized pyogranulomas, with a core of necrotic debris and viable and degenerate neutrophils surrounded by epithelioid macrophages, further bounded by a fibrous capsule and numerous lymphocytes and neutrophils. Occasionally, there were MNGC macrophages admixed with the epithelioid macrophages. Adjacent alveolar septa were often expanded by neutrophils and histiocytes, and alveolar, bronchiolar, and bronchial lumens were often expanded by a profound neutrophilic exudate. These lesions are similar to those described in melioidosis in humans [57]. Severe lung lesions in C57BL/6 mice were far less common, typically consisting only of interstitial inflammation and only rarely developing into well-organized pyogranulomas. By day 22 and beyond, significant lesions in the lung were completely lacking in C57BL/6 mice. This could be attributed to the purposefully low dose of bacteria used in this study, and it is possible that such a resolution of lung lesions would eventually have occurred in BALB/c mice; however we were unable to evaluate this as none of the mice survived beyond day 28.

**Fig 11:**
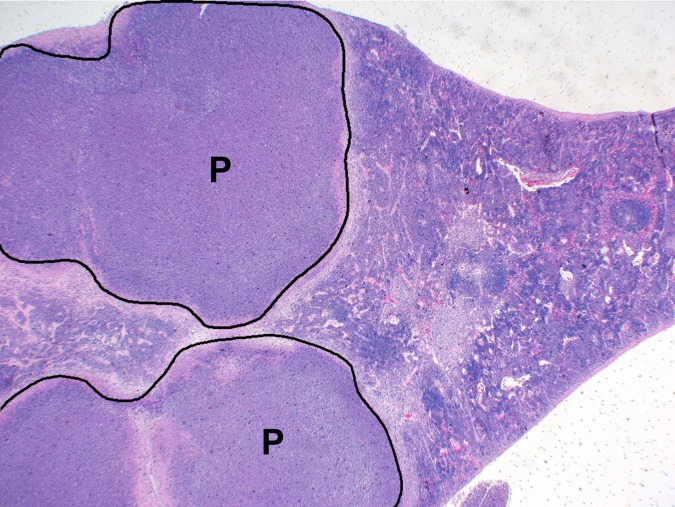
Spleen histopathology observed in mice following exposure to aerosolized *B*. *pseudomallei* K96243. BALB/c mouse euthanized on day 15 post-infection and displayed ruffled apperance at that time. Spleen: Multiple distinct pyogranulomas. P = pyogranuloma; H&E 20X.

In BALB/c mice, the spleen was another common location for development of acute suppurative and chronic granulomatous inflammatory lesions, affecting 12/29 mice. Early lesions starting on day 2 consisted of small foci of neutrophilic/suppurative inflammation with necrosis of adjacent red pulp elements and occasional fibrin thrombi. By day 15, inflammation progressed to histiocytic or pyogranulomatous inflammation, often with organized pyogranuloma formation similar to that seen in the lung (Fig 11). No such inflammatory lesions were noted in any of the C57BL/6 mice examined in this study.

In both strains of mice, liver involvement was not nearly as extensive as observed in the mice exposed via the IP route. In fact, none of the aerosol exposed mice (BALB/c or C57BL/6) developed chronic pyogranulomatous lesions in the liver. Liver lesions were limited to small foci of neutrophilic inflammation with or without hepatocyte necrosis and scattered throughout. Based on immunohistochemistry for *Burkholderia* spp. exopolysaccharide antigens (S1 Fig), it is likely that at least a portion of these lesions can be attributed to *Burkholderia*; however, the remainder of these lesions likely represent inflammation and necrosis secondary to other enterohepatic bacteria, commonly seen in a background lesion which is commonly encountered in mice [66].

None of the aerosol exposed mice from either strain developed debilitating/paralytic lesions in the spine and rear legs as was seen frequently with IP exposure; however three BALB/c mice did develop pyogranulomas in the tail. These likely represent lesions that developed from secondary embolic spread of the *B*. *pseudomallei* from distant sites or secondary to inoculation through broken skin in these areas. One BALB/c mouse developed pyogranulomas in the pancreas, also likely a sequel to embolic spread of the bacteria. All mice from both strains developed hyperplasia of the myeloid component of the bone marrow, as well as variable amounts of extramedullary hematopoiesis in the liver and spleen, representing increased tissue demand for leukocytes; however, these lesions were not nearly as intense as those seen in mice exposed via the IP route.

#### Immunological response associated with mice exposed to aerosolized bacteria

**Fig 12:**
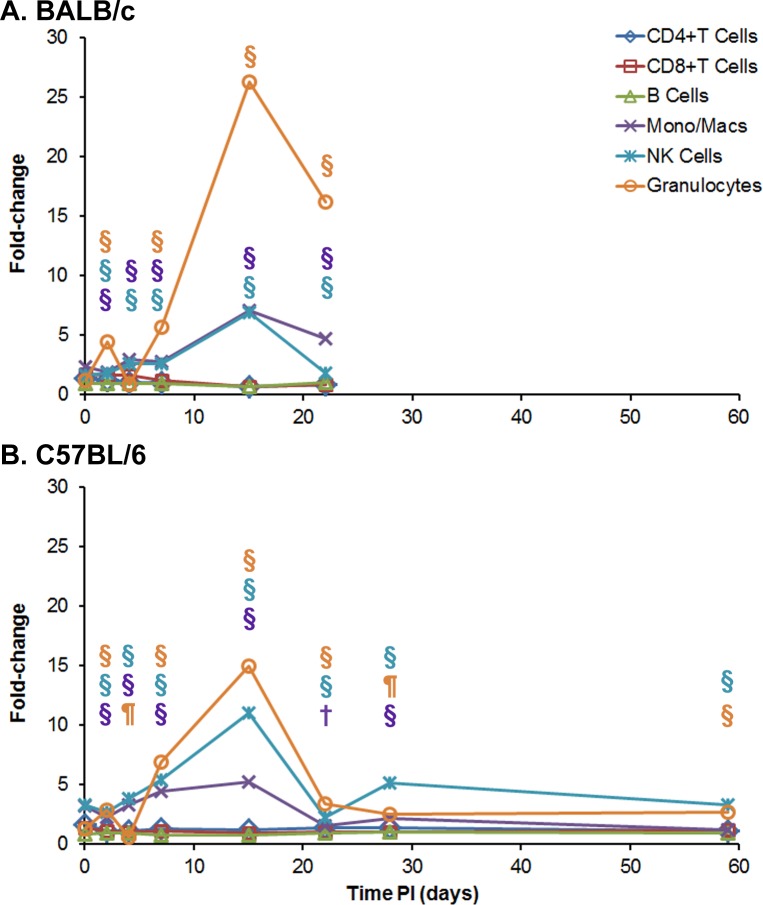
Cellular changes in spleens occurring in BALB/c and C57BL/6 mice after aerosol exposure to *B*. *pseudomallei* K96243. Spleen homogenates were prepared from infected **(A)** BALB/c and **(B)** C57BL/6 mice over time, and the percent of each cell type examined was determined. After 22 days there were no BALB/c mice survivors. For each mouse strain N = 5 at each time point. The fold-change for each cell type was determined by dividing the percent of the cell type at each time point (S6 Table) by the percent of the cell type present in normal, naïve mice, where N = 10 for BALB/c and N = 4 for C57BL/6 mice. Significant changes in % cell type (S6 Table) are shown above the inflammatory cells (macrophages/monocytes, NK cells, and granulocytes) only because they showed the most predominate changes through out the study. Significant levels compared to the naïve, control mice: † *P* < 0.05; ¶ *P* < 0.01; § *P* ≤ 0.001.

We also examined the cellular immune response in both BALB/c and C57BL/6 mice that were exposed to aerosolized *B*. *pseudomallei* K96243. We examined cellular changes that occurred in spleens from aerosol infected mice and cytokines/chemokines present in serum and expressed in spleen extracts from the same mice. All days described below represent days post exposure to *B*. *pseudomallei*. Fig 12 and S6 Table show the results of the analysis of the changes in cell composition of the spleens from the infected mice. However, unlike the IP exposure study described previously, *B*. *pseudomallei* K96243 aerosol exposed BALB/c mice did not survive beyond 22 days. As we saw in the IP challenge study, granulocytes (Ly6G+/CD44) were the predominant host cell that increased in the mouse spleen up to 22 days after aerosol exposure. At 2 days, we saw a small but significant transient (4.4-fold, *P* ≤ 0.001) increase in the granulocyte cell population that decreased on day 4 (0.85-fold), and then they increased to a maximum on day 15 in spleens from both BALB/c (26.3-fold, *P* ≤ 0.001) and C57BL/6 (15.0-fold, *P* *≤* 0.001) exposed mice (S6 Table and Fig 12). After this peak period, the number of granulocytes decreased in spleens from both mouse strains (16.2-fold, *P* ≤ 0.001; and 3.4-fold, *P* ≤ 0.001 respectively, at day 22). However, these values remained significantly above the levels in the naïve control mice. We also saw a significant increase in the other two types of inflammatory cells [monocytes/macrophages (CD11b+/CD14) and NK cells (CD49b/CD69)] in spleens at the same time in both strains of mice, except they did not reach as high a percentage as the granulocytes (S6 Table and Fig 12). In spleens from C57BL/6 mice at day 15, the number of NK cells rose to 11.0-fold (*P* ≤ 0.001) and in BALB/c we saw an increase in numbers to 6.9-fold (*P* ≤ 0.001) relative to the naïve control mice. In Fig 12 the fold changes in the number of monocytes/macrophages and NK cells were lower than that of the granulocytes because the initial amount (S6 Table) of granulocytes in naïve mice were much lower (0.89% in BALB/c and 0.56% in C57BL/6 mice) than that of monocytes/macrophages (4.38% in BALB/c and 2.75% in C57BL/6 mice) and NK cells (4.29% in BALB/c and 2.3% in C57BL/6 mice).

**Fig 13:**
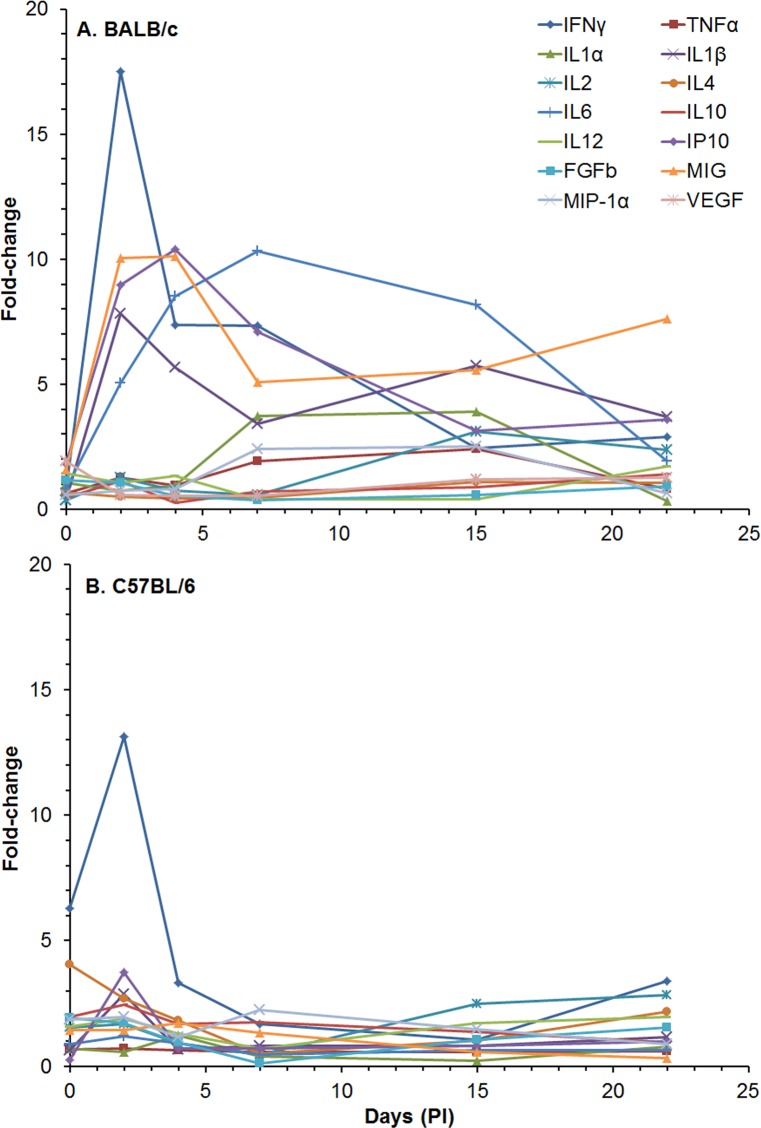
Changes in the amount of cytokines/chemokines in sera from BALB/c and C57BL/6 mice after aerosol exposure to *B*. *pseudomallei* K96243. The amount of cytokines/chemokines present in sera (S7 Table) was determined. For changes in cytokine/chemokine levels in sera from BALB/c mice **(A)**, we show changes in levels up to 22 days post exposure because there were no survivors after that period. We also show changes in cytokine/chemokine levels in sera for C57BL/6 mice **(B)** up to 22 days for comparison and not many significant changes occurred after 22 days in sera from C57BL/6 mice. For each mouse strain N = 5 at each time point. Fold-changes in cytokines/chemokines were determined by dividing the amount (pg/ml) present in sera of exposed mice (S7 Table) by the mount present in normal, naïve mice, where N = 10 for BALB/c and N = 4 for C57BL/6 mice. For C57BL/6 mice fold-change for MIG was not shown because it was very high (235-fold), and it would make obscure the changes in the levels of the other cytokines/chemokines at the same time.

We then examined the levels of cytokines/chemokines present in the sera of aerosol exposed BALB/c and C57BL/6 mice (S7 Table). In Fig 13, we showed the changes in the cytokines/chemokines in sera for this study up to 22 days for C57BL/6 because there were no BALB/c survivors after that time for comparison, and there were not many significant changes in the cytokine/chemokine levels in sera from C57BL/6 mice after 22 days post exposure. The amount of cytokines/chemokines in sera after 28, 59, and 91 days after exposure to bacteria in C57BL/6 mice can be seen in S7 Table and S5 Fig. Immediately after the mice were exposed to *B*. *pseudomallei* K96243 (day 0, 4–6 h post exposure) we detected a significant rise in IFN-γ [68.6 (1.06) pg/ml, *P* < 0.05], IL-4 [134.4 (1.14) pg/ml, *P* < 0.05], IL-10 [85.4 (1.13) pg/ml, *P* < 0.01], FGFb [507.2 (1.03) pg/ml, *P* < 0.05] and MIG [133.6 (1.22) pg/ml, *P* < 0.01] in sera from C57BL/6mice (S7 Table). After 2 days, we saw a significant increase in more cytokines/chemokines in sera from both mouse strains (S7 Table and Fig 13). Overall, from 4 days to 22 days, we detected an increase in more cytokines/chemokines in sera from BALB/c mice than from C57BL/6 (S7 Table and Fig 13). Those would include IFN-γ, IL-1α, IL-1β, IL-6, IP-10, and MIG. We did not show the fold-change of the chemokine MIG in sera from C57BL/6 in Fig 13 because it was very high at 2 days (235-fold), and it would make it difficult to see the changes in the amounts of the other cytokines/chemokines in the same figure. From day 28 to 91 days, we saw significantly increased levels of IL-2 and MIG in sera from C57BL/6 mice (S7 Table).

**Fig 14:**
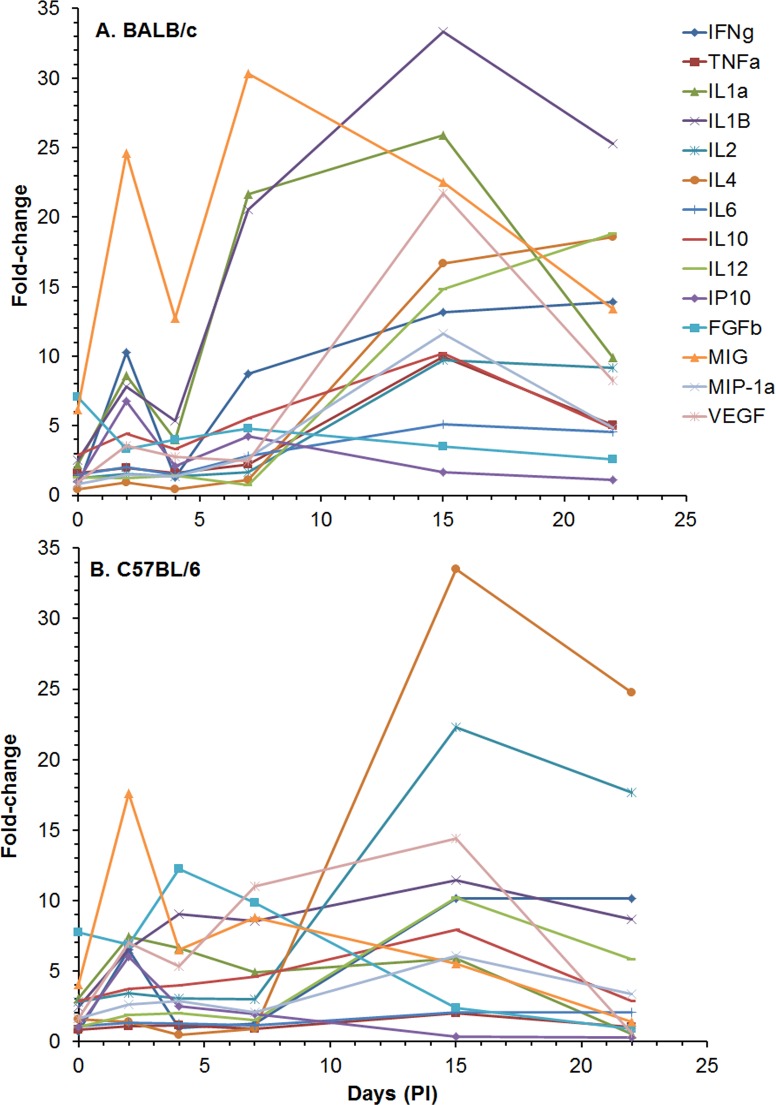
Changes in the amount of cytokines/chemokines in spleen extracts from BALB/c and C57BL/6 mice after aerosol exposure to *B*. *pseudomallei* K96243. The amount of cytokines/chemokines present in spleen extracts (S8 Table) was determined. For changes in cytokine/chemokine levels in spleen extracts from BALB/c mice **(A)**, we show changes in levels up to 22 days because there were no survivors after that period. We also show changes in cytokines/chemokine levels in spleen extracts for C57BL/6 mice **(B)** for comparison although they were determined up to day 91 (S8 Table). For each time point, N = 5 for BALB/c and C57BL/6 mice. Fold-changes in cytokines/chemokines was determined by dividing the amount (S8 Table) present in the spleen extract by the amount present in normal, naïve mice, where N = 10 for BALB/c and 4 for C57BL/6 mice.

We also examined the cytokines/chemokines present in the spleen extracts from the BALB/c and C57BL/6 mice that were exposed to *B*. *pseudomallei* K96243 by aerosol (S8 Table and Fig 14, data through day 91 are depicted in S5 Fig). We saw many more immediate (day 0, 4–6 h post exposure) increases in cytokines/chemokines levels in the spleen homogenates of both stains of mice than we saw in sera (S8 Table). We saw a significant increase in the expression of many of the cytokines/chemokines after 2 days in both mouse strains, but the fold-change was more apparent in spleen extracts from BALB/c mice (Fig 14). MIG levels again showed the largest and rapid changes early after infection in spleen extracts from both mice. In BALB/c mice we detected a rapid rise up to 2 days [3494 (1.36) pg/ml, *P* ≤ 0.001] before it decreased at day 4 [1813 (1.19) pg/ml, *P* ≤ 0.001), and MIG levels peaked at 7 days [4313 (1.16) pg/ml, *P* ≤ 0.001) before there was a gradual decrease to 22 days [1905 (1.50) pg/ml, *P* < 0.01) after which all BALB/c mice had succumbed to disease or were euthanized. We also detected a large increase in the inflammatory cytokines IL-1α and IL-1β in spleen homogenates from BALB/c mice that peaked at 15 days [1963 (3.28) pg/ml and 1296 (1.45) pg/ml (*P* ≤ 0.001), respectively] before gradually decreasing to day 22 (S8 Table and Fig 14A). Although we saw increases in these two inflammatory cytokines in spleen extracts from C57BL/6 mice, they did not reach levels seen in BALB/c mice (S8 Table and Fig 14B). One cytokine that we found high levels present in spleen extracts from both mice was IL-4, which is a T-helper type 2 (Th2) cytokine, although more was present in C57BL/c spleen extracts where levels peaked at 28 days [522.2 (1.07) pg/ml, *P* ≤ 0.001] before it decreased to basal levels at 59 and 91 days (S8 Table). We also saw high levels of IFN-γ in spleen extracts from BALB/c mice at 2 days and 7 to 22 days, but at 2 days and 15 to 28 days in extracts from C57BL/6 mice. IL-2 had the second highest fold-change in spleen extracts from C57BL/c mice that peaked at 15 days. Both IFN-γ and IL-2 are considered Th1-type cytokines. Overall, we detected more cytokines/chemokines in spleen extracts from BALB/c mice than from C57BL/6 mice by day 15 and generally at higher levels (S8 Table and Fig 14). The BALB/c mice sampled on day 22 displayed high levels of IL-1α, IL-1β, IL-2, IL-4, IL-12, IFN-γ, MIG, and TNF-α in spleen homogenates. In addition, there were at least two general peaks of cytokines/chemokines activity in spleen extracts we observed from both mice that occurred at 2 days and 15 days. Finally, we saw a mixed Th1- and Th2-like cytokine production in spleen extracts from both BALB/c and C57BL/6 mice.

#### Differences in adaptive immune response observed between strains of mice and different routes of infection

Sera were used to perform ELISAs designed to determine antibody titers directed against irradiated-inactivated *B*. *pseudomallei* K96243 cells. As demonstrated in S9 Table, the antibody response varied widely in the different mice regardless of the challenge route. These data suggested that after IP infection C57BL/6 produced more antibodies (*P* < 0.001) early after infection (day 7). The BALB/c mice exposed to aerosolized bacteria demonstrated trends suggesting an overall more robust antibody response (*P* < 0.01 and < 0.001 on days 14 and 21 after exposure to aerosolized *B*. *pseudomallei*, respectively). This observation emphasizes the Th2 response typically associated with BALB/c mice but could also be due in part to the greater number of LD_50_ equivalents delivered to the BALB/c mice compared to the C57BL/6 mice (exposed to similarly low CFU but approximately 4 times greater LD_50_ equivalents).

#### The presence of Multi-Nucleated Giant Cells (MNGCs) is appreciable in mice exposed to aerosolized bacteria

**Fig 15:**
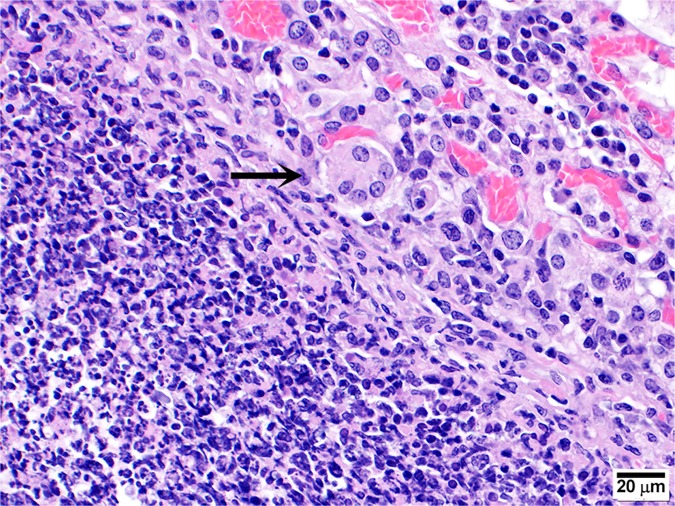
Representative micrograph demonstrating the presence of MNGC in mice exposed to aerosolized bacteria. MNGC (arrow) observed in the lungs of a BALB/c mouse exposed to approximately 5 CFU of aerosolized *B*. *pseudomallei* K96243 and was euthanized after meeting early end-point euthanasia criteria 20 days post-exposure.

MNGCs were not readily observed in the animals challenged by the IP route in this study. This was in contrast to data presented by Chirakul et al. that indicated that MNGCs were present in the spleens of BALB/c mice challenged IP with *B*. *pseudomallei* K96243 [55]. Previous reports of MNGC macrophages in chronic melioidosis suggest that these cells may be seen in a second wave of inflammation, perhaps from a recrudescence of *B*. *pseudomallei* infection [55, 57]. It is possible that if mice were sacrificed at later time points (> 60 days post-infection), such lesions might be more prominent in the mice challenged via the IP route. We did observe occasional MNGCs in the lungs of 3/28 C57BL/6 mice and both the lungs and spleens of 9/29 BALB/c mice exposed to aerosolized *B*. *pseudomallei* (Fig 15).

## Discussion

We systematically characterized the disease progression after introduction of *B*. *pseudomallei* K96243 into either BALB/c or C57BL/6 mice by either IP injection or by exposure to aerosolized bacteria. Our data support and expand upon other work [23, 27, 31–40, 42–45] suggesting that each strain of mouse has strengths and weaknesses when studying the pathogenesis of *B*. *pseudomallei* and as models for the human responses to infection. To our knowledge, this current work is among the most comprehensive reports of these murine disease models, due to our time course and extensive sample collection to include pathologic and immune analyses. While the ultimate goal of biodefense research is to elucidate therapies and vaccines to treat inhalational forms of these diseases, it is important to also have a well-characterized alternate model due to logistic and financial constraints when dealing with aerosol exposure studies. We included the IP model as an alternate for several reasons, i.e. ease and reproducibly of the exposure methodology, and the fact that many of the clinical signs may potentially mimic some of those observed in human cases of melioidosis [36, 45]. As illustrated in Fig 2, the dissemination patterns after IP injection of the bacteria were fairly similar between BALB/c and C57BL/6 mice even considering the disparity between the numbers of bacteria administered to achieve comparable LD_50_ equivalents in each mouse strain. While the amount of bacteria used to challenge the C57BL/6 mice was approximately 30 times greater than the challenge dose used for BALB/c mice, the resulting levels of bacteremia were similar. This clearly confirms that the C57BL/6 immune response is better suited to combat infection with *B*. *pseudomallei* K96243. Interestingly, the bacteria can disseminate to the lungs very early after IP injection, but by day 4 post-infection the majority of lungs were free of bacteria.

These data collected from mice exposed to aerosolized bacteria demonstrated a similar trend. We attempted to deliver comparable LD_50_ equivalents by exposing mice to aerosolized bacteria. However, due to the difficulty associated with reproducibly delivering very low doses of bacteria (i.e. < 20 CFU), the BALB/c mice received approximately 0.2 LD_50_ equivalent (5 CFU) whereas the C57BL/6 mice received approximately 0.05 LD_50_ equivalents (18 CFU). This 4-fold difference in achieved delivered LD_50_ equivalents may account for some of the differences we observed. When mice were exposed to aerosolized bacteria, there was an increase in bacterial burdens in either strain of mouse; however the extent and duration of detectable bacteria in tissues were appreciably greater and longer lasting in BALB/c as compared to C57BL/6 mice. C57BL/6 mice were never observed to be bacteremic, whereas BALB/c mice were demonstrably bacteremic from day 4 through day 15. In the case of lung bacterial burden, C57BL/6 mice experienced bacterial replication through day 4, followed by a decline, and then plateau of bacterial growth. The BALB/c mice exhibited a greater and longer lived bacterial burden in the lungs. The bacterial CFU data obtained from the spleen homogenates were noteworthy. The BALB/c mice exposed to aerosolized bacteria had rapid and robust dissemination to and replication within the spleen, which peaked at approximately day 7 but remained significant throughout the entire study. The C57BL/6 mice exposed to aerosolized bacteria, however, demonstrated rapid dissemination, but replication peaked at day 2 and then bacterial growth was not observed in the spleen tissues after day 7. Interestingly, however, bacteria were detected in samples collected on day 91 post exposure to aerosolized bacteria, lending further support to the concept that C57BL/6 mice may represent an appropriate chronic disease model. In our studies, particles containing aerosolized bacteria were approximately 1–3 μm in diameter. There is evidence that different particle sizes can lead to different disease progression. Thomas et al. demonstrated that *B*. *pseudomallei* delivered in larger droplets (i.e. 12 μm in diameter) resulted in a greater involvement in the nasal tissues, olfactory mucosa, olfactory nerve, olfactory bulb and brain tissue [68]. Similar results have been demonstrated when mice were exposed to *B*. *pseudomallei* via intranasal instillation which inherently delivers large droplets of bacteria [69]. This was in contrast to pathology observed when mice were exposed to 1 μm sized particles of aerosolized bacteria which displayed more significant lung pathology, as well as involvement of the mediastinal lymph nodes [68]. The impact of particle size on *B*. *pseudomallei* pathogenesis has also been established in intratracheal instillation of *B*. *pseudomallei* when separate laboratories utilized similar techniques but their respective equipment yielded differing particle sizes [70, 71].

Interestingly, we observed MNGCs in both the spleen and lungs of BALB/c and in lungs of C57BL/6 mice after exposure to aerosolized bacteria (Fig 15). We were unable to identify MNGCs in either mouse strain following the introduction of bacteria via IP injection. Again, these differences underscore the importance of bacterial strain, delivered dose, and delivery route. Reports suggest a correlation of MNGC formation with other virulence attributes [72], and our previous work has proposed a frequent inverse correlation between bacterial growth and cytotoxicity activity (to include the number and size of MNGC formation) in an *in vitro* macrophage model with virulence in mouse models of infection [36]. Chirakul et al. reported differences in the inflammatory response observed in BALB/c mice compared to C57BL/6 mice when infected with either wild-type *B*. *pseudomallei* K96243 or a mutant strain with altered type III secretion system expression [55]. These authors found MNGC formation in BALB/c mice infected with either strain of *B*. *pseudomallei* and demonstrated that bacterial genes (i.e. *bprD*) may be expressed differentially in BALB/c mice compared to C57BL/6 mice [55]. Why the MNGCs were encountered in the aerosol challenged mice, and not the IP exposed mice is unclear. Perhaps initial passage through alveolar macrophages early in the disease process enhances the ability of the bacterial to induce MNGC formation or possibly the IP exposure in some way reduces this ability. Alternately, the differences could be attributed to study-specific parameters (i.e. bacterial strain or doses delivered). Further studies specifically examining MNGC prevalence *in vivo* and potential significance of such altered cellular morphologies are warranted.

Although histochemical analysis of infected tissue identified the infiltrating granulocytes were predominately neutrophils, we also used flow cytometry to identify that the major infiltrating cells into the infected spleens were Ly6G+, infiltrating monocytes/macrophages and NK cells. Although there were similarities in the temporal pattern and the amount of infiltrating inflammatory cells into the spleens in these two mouse strains after IP infection, there were some differences in the overall immune response and susceptibility of BALB/c mice to *B*. *pseudomallei* K96243 infection compared to the more resistant C57BL/6 mice. In the cellular innate immune response in the IP challenge study, there was a transitory increase in Ly6G+ granulocytes, monocytes/macrophages, and NK cells between 2–7 days after infection in C57BL/6 mice that was not evident in BALB/c mice that were infected by the same route. In the aerosol challenge study, there was a minor but appreciable transitory increase in granulocytes in both mouse strains was observed but not with the other inflammatory cells two days after infection. It is not clear at this time if the early transitory peak of granulocytes reflects an initial response to CFU appearing in the spleen or an initial infiltration of granulocytes that contain *B*. *pseudomallei* that appear in the spleen within 1–2 days after infection, although this early association with Ly6G+ neutrophils was also previously observed in lungs of BALB/c mice [73]. Although we cannot discount the influence of the number of CFU used between the IP and aerosol challenge study, one of the major differences between the two routes of infection was the larger number of Ly6G+ granulocytes that infiltrated the spleens of the two mouse strains by the IP route over that by the aerosol route of infection. At the same time, the number of the other innate immune inflammatory cells (monocytes/macrophages and NK cells) did not appear to appreciably change over the same period. A number of reports note the importance of neutrophils in the response to or required for clearance of *B*. *pseudomallei* in BALB/c or C57BL/6 mice [37, 45, 73–75]. The peak level of Ly6G+ granulocytes present in spleens of both BALB/c and C57BL/6 mice challenged by the IP route was between 15 to 22 days with the maximum at day 22 (36.6- and 39-fold increase, respectively), which was also similar to the peak levels that occurred for monocytes/macrophages and NK cells in the same spleens, but they were present in much lower amounts. At the same time, the CFU load in the various organs and blood was decreasing or close to the limit of detection. At the end of the IP challenge study (59 days), we saw a significant fold increase in monocytes/macrophages, granulocytes, and NK cells, with former cells being the most abundant at day 59. In the aerosol challenged mice, however, the maximum level of Ly6G+ granulocytes in spleens was detected at 15 days in both BALB/c and C57BL/6 mice (25.5- and 17.2-fold increase, respectively), but the peak amounts were lower than detected in the IP challenged mice. After 22 days in the spleens of aerosol infected BALB/c mice, the amount of Ly6G+ granulocytes was still 15.5-fold over the control naïve mice levels, while in the spleens of C57BL/6 mice they were down to 3.4-fold over the control mice. This may be because the spleens of the same BALB/c mice on day 22 still has a modest number of CFU (geometric mean > 100 CFU/g), while spleens from C57BL/6 mice had barely detectable numbers of CFU at the same time (Fig 8).

Besides the differences that we noted in the expression of cytokines/chemokines between strains of mice, there were also differences in cytokine expression which were route-associated. In serum of BALB/c mice that were exposed to aerosolized bacteria, we detected more cytokines/chemokines present for up to 22 days post exposure than we do in sera from mice that were infected by the IP route. We saw heightened levels of IFN-γ, IP-10, IL-1β, IL-6, and we detected longer expression of MIG over the period measured in the aerosol exposed BALB/c mice. We detected some cytokines/chemokines in the sera of BALB/c mice infected by the IP route, such as KC and IL-1α that appeared to be immediately expressed upon exposure to *B*. *pseudomallei* K96243. KC is a chemokine that is a major chemoattractant for neutrophils, and it has been suggested that it may be a homolog of the human IL-8 from its ability to bind to a murine IL8 type B receptor [76]. IL-1α and IL-1β are proinflammatory cytokines that are the host’s innate immune response to exposure to the pathogen. One common phenomenon we observed in sera from C57BL/6 mice that were challenged by either IP or aerosol at day 2 was an enormous, transient increase of MIG that we did not observe in sera of *B*. *pseudomallei* K96243 infected BALB/c mice by either route. It went up to 235-fold above what was normally seen in naïve mice in both cases. MIG and IP-10, the latter which was also elevated, belong to the CXCL family of chemokines, CXCL9 and CXCL10, respectively, which are both induced by IFN-γ and share the same receptor CXCR3 (as well as CXCL11 or I-TAC) [77]. The expression of CXCL9 (MIG) can be detected in many antigen presenting cells, and the receptor CXCR3 is present on activated T cells and B cells that may influence both cellular immunity and antibody responses to the presence of a pathogen [78, 79]. The expression of IP-10, in addition, can also be induced by IFN-α and IFN-β [78], and it can be secreted by monocytes, endothelial cells, fibroblasts, and keratinocytes [80]. In our present study, we observed more IFN-γ in serum from both BALB/c and C57BL/6 mice that were exposed to *B*. *pseudomallei* K96243 by aerosol than the IP infected mice, and although we saw a higher peak of MIG in sera (at day 2) from IP infected BALB/c mice, the elevated levels of MIG and IP-10 in sera from aerosol exposed mice were observed for a longer period after infection. Elevated levels of IP-10 and MIG have been observed in severe human melioidosis cases on admission in a clinical setting and during antibiotic treatment [81].

This abundance of MIG and IP-10, which are chemoattractants of activated T-cells [78, 82], may suggest that T-cells are involved in the infection, pathogenesis, and immunity to *B*. *pseudomallei*, although in this study we did not examine the activity of T cells [78, 82–86]. In IP infected mice IL-1α and IL-1β were the predominant innate immune inflammatory cytokines that were more apparent than in aerosol infected mice. IL-1β, which appears to be generated by a special cytosolic inflammasome and is a deleterious cytokine, is the more prevalent of the two cytokines [74, 87, 88]. Finally, there appeared to be a mixed cytokine response with Th1- and Th2-like cytokines expressed in response to *B*. *pseudomallei* in the murine models even in the Th-1-like C57BL/6 mouse after aerosol infection. This immune response by the host may be partly responsible for the inability of the host to completely resolve the infection and could lead to a fatal outcome in both acute and chronic infections by *B*. *pseudomallei*.

The differences and similarities we highlighted here are important; however, we do not want to oversimplify or understate the complex process of selecting the appropriate model for melioidosis. Inherent differences between BALB/c and C57BL/6 mice are numerous and well documented. Whereas BALB/c mice mount a rapid and robust Th-2 like response, their adaptive Th-1 response is not as efficient nor long lasting when compared to that of C57BL/6 mice [89–94]. The differential immune responses have been observed to include cellular recruitment kinetics and downstream cellular functionality (i.e. cytokine and chemokine expression) [27, 34, 95]. Accordingly, BALB/c mice are known to be more susceptible to autoimmune disease [96–98] and are more susceptible to tumor proliferation in certain models [99–101]. BALB/c mice and macrophages derived from these mice are also well documented to be more susceptible to infectious diseases (to include bacteria, intracellular bacteria/parasites, and viruses) [32, 41, 92, 94, 102–112]. Thus, there are many factors that need to be taken into account when determining applicability of a mouse model and subsequently how to analyze these data from said models [91, 104, 113]. Additionally, when specifically examining *B*. *pseudomallei* the bacterial strain selection and route of infection are of the utmost importance. The virulence of the bacterial strains are known to vary substantially [36, 41, 45, 94, 102–104, 114], and the route of infection can significantly alter the disease pathogenesis as well. In conclusion, the BALB/c and C57BL/6 mouse each model different parameters of melioidosis. BALB/c mice may be more appropriate for virulence testing/characterization of bacterial strains, and C57BL/6 may be best suited for vaccine or therapeutic testing, and perhaps when used together result in a more complete understanding of bacterial pathogenesis and efficacy testing of medical counter-measures.

## Supporting information

S1 Fig Representative immunohistochemistry of selected tissues. **(A)** BALB/c mouse exposed 5 CFU of aerosolized *B*. *pseudomallei* and euthanized 20 days post-exposure exhibiting spleen pyogranulomas; 40X; **(B)** BALB/c mouse challenged with 3 x 10^4^ CFU of *B*. *pseudomallei* by the IP route and euthanized 2 days post-exposure exhibiting liver inflammation and necrosis; 40X **(C)** C57BL/6 mouse exposed to 18 CFU of aerosolized *B*. *pseudomallei* and euthanized 10 days post-exposure exhibiting lung pneumonia; 100X **(D)** BALB/c mouse exposed to 5 CFU of aerosolized *B*. *pseudomallei* and euthanized 7 days post-exposure exhibiting lung pyogranuloma; 100X **(E)** BALB/c mouse exposed to 5 CFU of aerosolized *B*. *pseudomallei* and euthanized 10 days post-exposure exhibiting otitis media; 100X and **(F)** BALB/c exposed to 5 CFU of aerosolized *B*. *pseudomallei* and euthanized 4 days post-exposure exhibiting inflammation in nasal cavity; 100X.
(TIF)

S2 Fig Spleen weights of mice following IP challenge with *B*. *pseudomallei* K96243. As observed previously, spleen weight can be indicative of intrinsic diffrences in host immune response or bacterial replications [36, 45]. After IP infection with similar LD_50_ equivalents, trends in spleen weight in both BALB/c and C57BL/6 mice were comparable, except on day 4 where C57BL/6 mice spleens were significantly larger than BALB/c mice mice (*P* = 0.0122).
(TIF)

S3 Fig Spleen weights of mice following exposre to aerosolized *B*. *pseudomallei* K96243. As observed previously, spleen weight can be indicative of intrinsic diffrences in host immune response or bacterial replications [36, 45]. After exposre to low doses the spleens harvested from BALB/c mice were signifcantly larger on days 15 and 22 post exposure (*P* = 0.0324 and 0.0007, respectively).
(TIF)

S4 Fig Changes in the amount of cytokines/chemokines in sera from BALB/c and C57BL/6 mice after IP infection with *B*. *pseudomallei* K96243. The amount of cytokines/chemokines present in sera (S3 Table) from infected **(A)** BALB/c and **(B)** C57BL/6 mice was determined. The fold-change in MIG levels in sera was not shown for C57BL/6 because it was very high at 2 days post-infection (235-fold), and it would make it difficult to see changes in other cytokines/chemokines for comparison. For each time point, N = 5 for BALB/c and C57BL/6 mice. Fold-change in cytokines/chemokines was determined by dividing the amount (pg/ml) present in sera after infection by the amount present in normal, naïve mice, where n was 10 for BALB/c and N = 4 for C57BL/6 mice.
(TIF)

S5 Fig Changes in the amount of cytokines/chemokines in sera and spleen from C57BL/6 mice after aerosol exposure to *B*. *pseudomallei* K96243 through day 91 post exposure to aerosolized bacteria. The amount of cytokines/chemokines present in sera (S7 Table) was determined. For changes in cytokine/chemokine levels in sera from C57BL/6 mice **(A)**, we show changes in levels up to 91 days post-infection. We also show changes in cytokine/chemokine levels in spleen extracts for C57BL/6 mice **(B)** up to 91 days post-infection for comparison. For each mouse strain N = 5 at each time point. Fold-changes in cytokines/chemokines were determined by dividing the amount (pg/ml) present in sera of exposed mice (S7 Table) by the mount present in normal, naïve mice, where n was 10 for BALB/c and 4 for C57BL/6 mice. For C57BL/6 mice fold-change for MIG was not shown because it was very high (235-fold), and it would make it difficult to see the changes in the levels of the other cytokines/chemokines at the same time.
(TIF)

S1 Table Temperatures and body weights recoded daily after IP challenge with *B*. *pseudomallei* K96243. (XLSX)

S2 Table Cellular changes in spleen composition in BALB/c and C57BL/6 mice after IP challenge with *B*. *pseudomallei* K96243. (XLSX)

S3 Table Cytokines/chemokines in serum from BALB/c and C57BL/6 mice after IP challenge with *B*. *pseudomallei* K96243. (XLSX)

S4 Table Cytokines/chemokines in spleen extracts from BALB/c and C57BL/6 mice after IP challenge with *B*. *pseudomallei* K96243. (XLSX)

S5 Table Temperatures and body weights recoded daily after exposure to aerosolized *B*. *pseudomallei* K96243. (XLSX)

S6 Table Cellular changes in spleen composition in BALB/c and C57BL/6 mice after exposure to aerosolized *B*. *pseudomallei* K96243. (XLSX)

S7 Table Cytokines/chemokines in sera from BALB/c and C57BL/6 mice after exposure to aerosolized *B*. *pseudomallei* K96243. (XLSX)

S8 Table Cytokines/chemokines in spleen extracts from BALB/c and C57BL/6 mice after exposure to aerolized *B*. *pseudomallei* K96243. (XLSX)

S9 Table Anti-*B*. *pseudomallei* antibody titers in BALB/c and C57BL/6 mice at select points after exposure to *B*. *pseudomallei*. (XLSX)
