# Supplementary figures and images for: Characterization of pathogenesis of and immune response to Burkholderia pseudomallei K96243 using both inhalational and intraperitoneal infection models in BALB/c and C57BL/6 mice
Source: PLoS One. 2017 Feb 24;12(2):e0172627. doi: 10.1371/journal.pone.0172627 (PMC5325312; doi:10.1371/journal.pone.0172627)

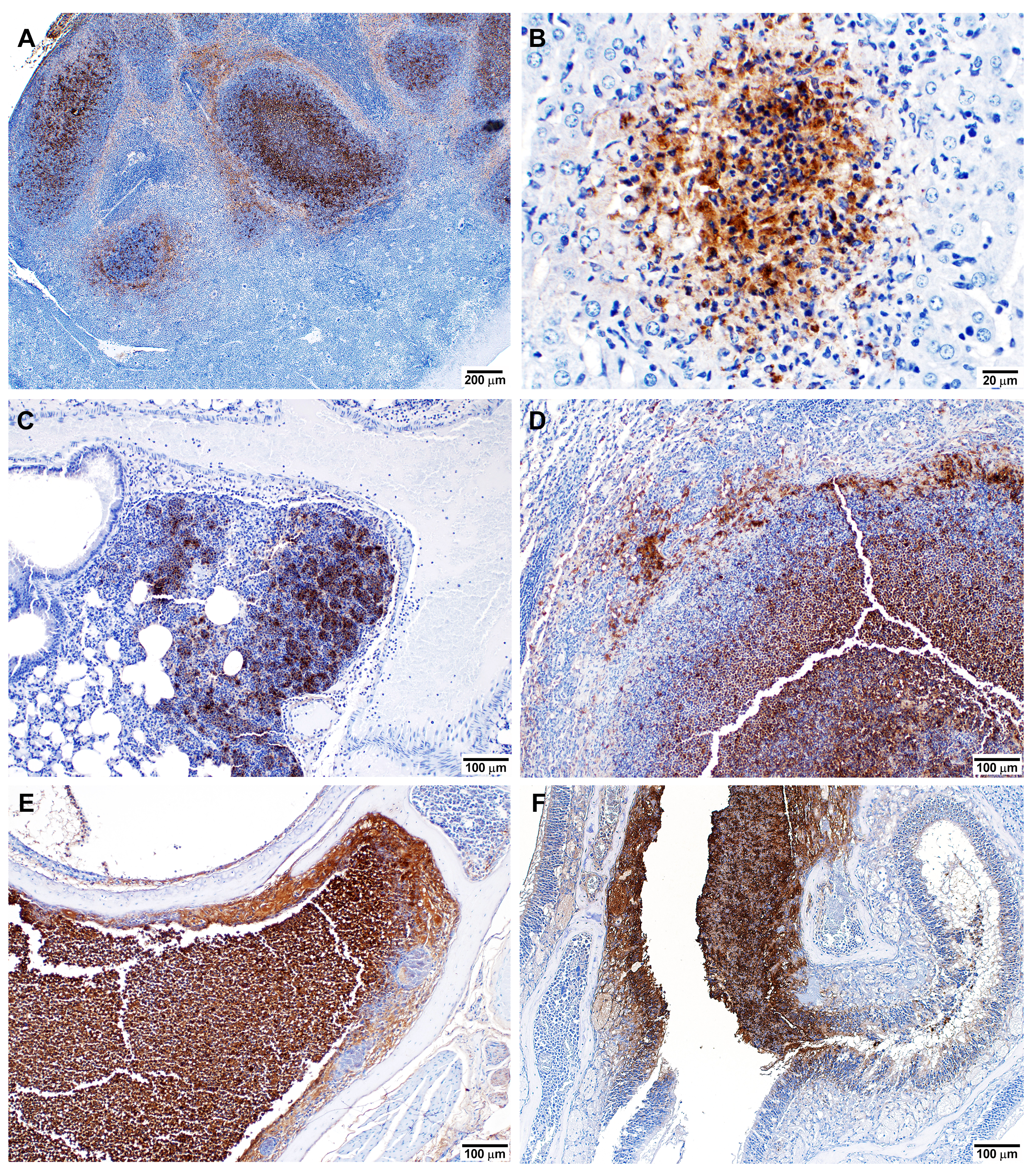

Supplement: S1 Fig — (A) BALB/c mouse exposed 5 CFU of aerosolized B. pseudomallei and euthanized 20 days post-exposure exhibiting spleen pyogranulomas; 40X; (B) BALB/c mouse challenged with 3 x 10^4^ CFU of B. pseudomallei by the IP route and euthanized 2 days post-exposure exhibiting liver inflammation and necrosis; 40X (C) C57BL/6 mouse exposed to 18 CFU of aerosolized B. pseudomallei and euthanized 10 days post-exposure exhibiting lung pneumonia; 100X (D) BALB/c mouse exposed to 5 CFU of aerosolized B. pseudomallei and euthanized 7 days post-exposure exhibiting lung pyogranuloma; 100X (E) BALB/c mouse exposed to 5 CFU of aerosolized B. pseudomallei and euthanized 10 days post-exposure exhibiting otitis media; 100X and (F) BALB/c exposed to 5 CFU of aerosolized B. pseudomallei and euthanized 4 days post-exposure exhibiting inflammation in nasal cavity; 100X. (TIF) [file pone.0172627.s001.tif]

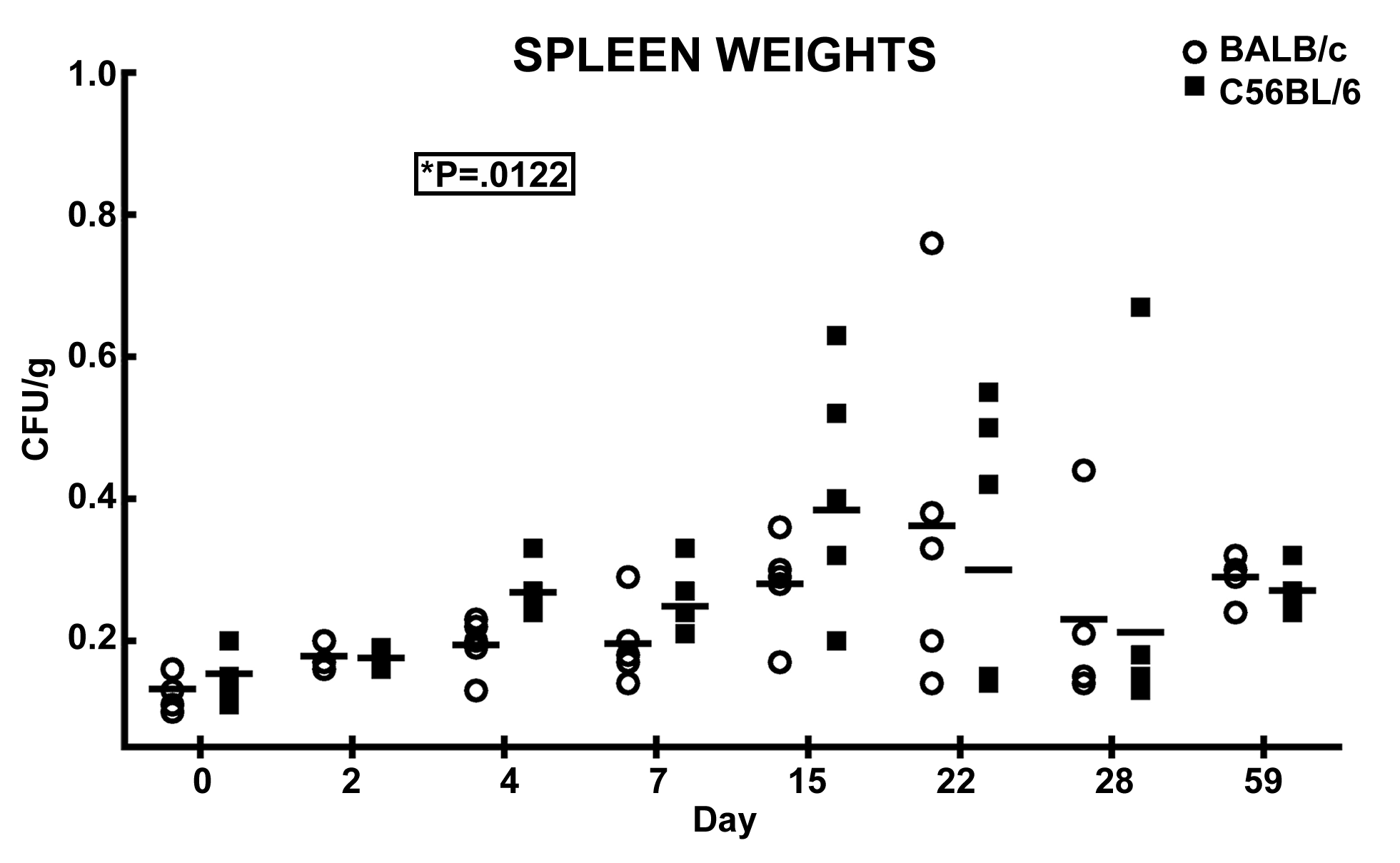

Supplement: S2 Fig — As observed previously, spleen weight can be indicative of intrinsic diffrences in host immune response or bacterial replications [36, 45]. After IP infection with similar LD50 equivalents, trends in spleen weight in both BALB/c and C57BL/6 mice were comparable, except on day 4 where C57BL/6 mice spleens were significantly larger than BALB/c mice mice (P = 0.0122). (TIF) [file pone.0172627.s002.tif]

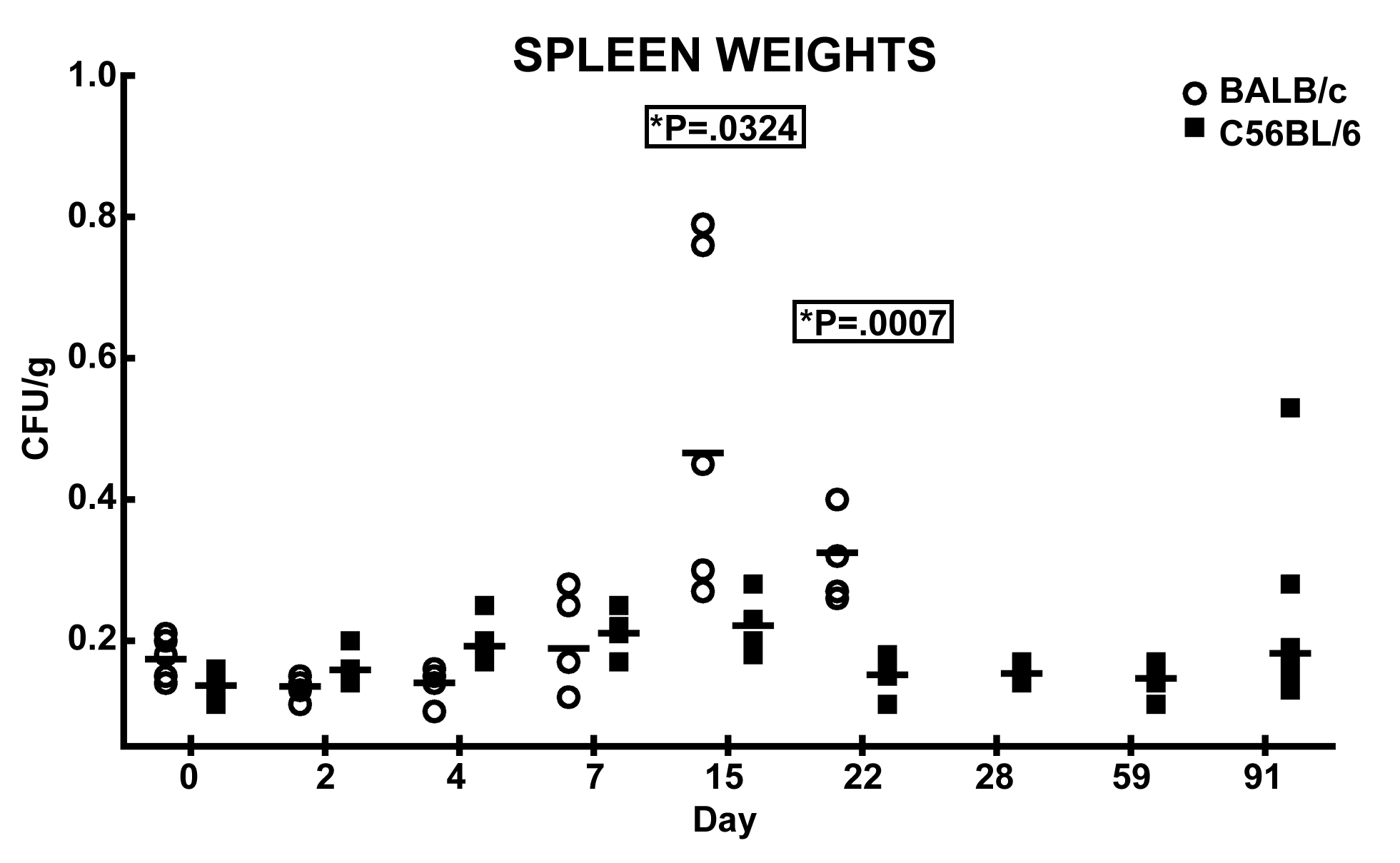

Supplement: S3 Fig — As observed previously, spleen weight can be indicative of intrinsic diffrences in host immune response or bacterial replications [36, 45]. After exposre to low doses the spleens harvested from BALB/c mice were signifcantly larger on days 15 and 22 post exposure (P = 0.0324 and 0.0007, respectively). (TIF) [file pone.0172627.s003.tif]

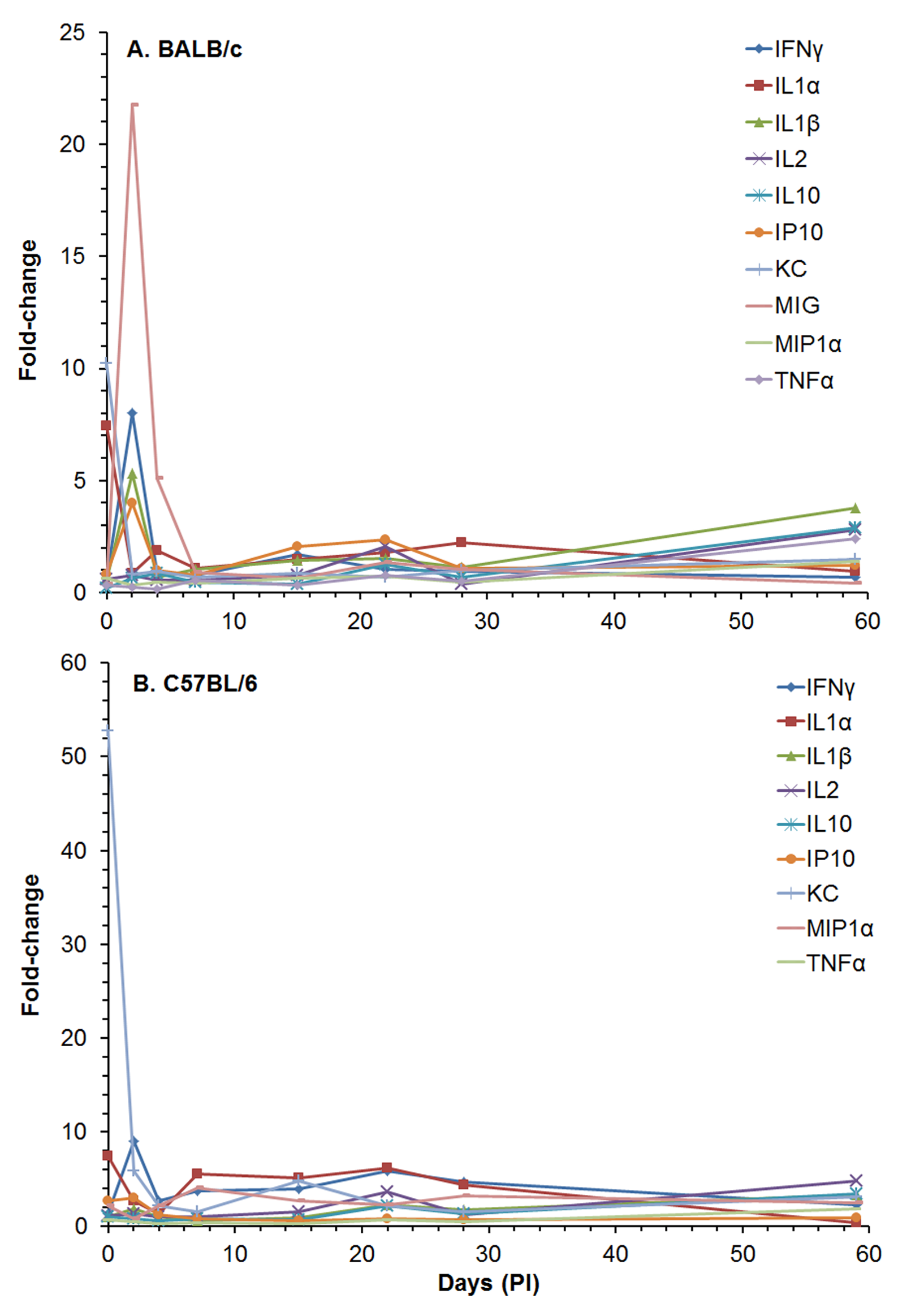

Supplement: S4 Fig — The amount of cytokines/chemokines present in sera (S3 Table) from infected (A) BALB/c and (B) C57BL/6 mice was determined. The fold-change in MIG levels in sera was not shown for C57BL/6 because it was very high at 2 days post-infection (235-fold), and it would make it difficult to see changes in other cytokines/chemokines for comparison. For each time point, N = 5 for BALB/c and C57BL/6 mice. Fold-change in cytokines/chemokines was determined by dividing the amount (pg/ml) present in sera after infection by the amount present in normal, naïve mice, where n was 10 for BALB/c and N = 4 for C57BL/6 mice. (TIF) [file pone.0172627.s004.tif]

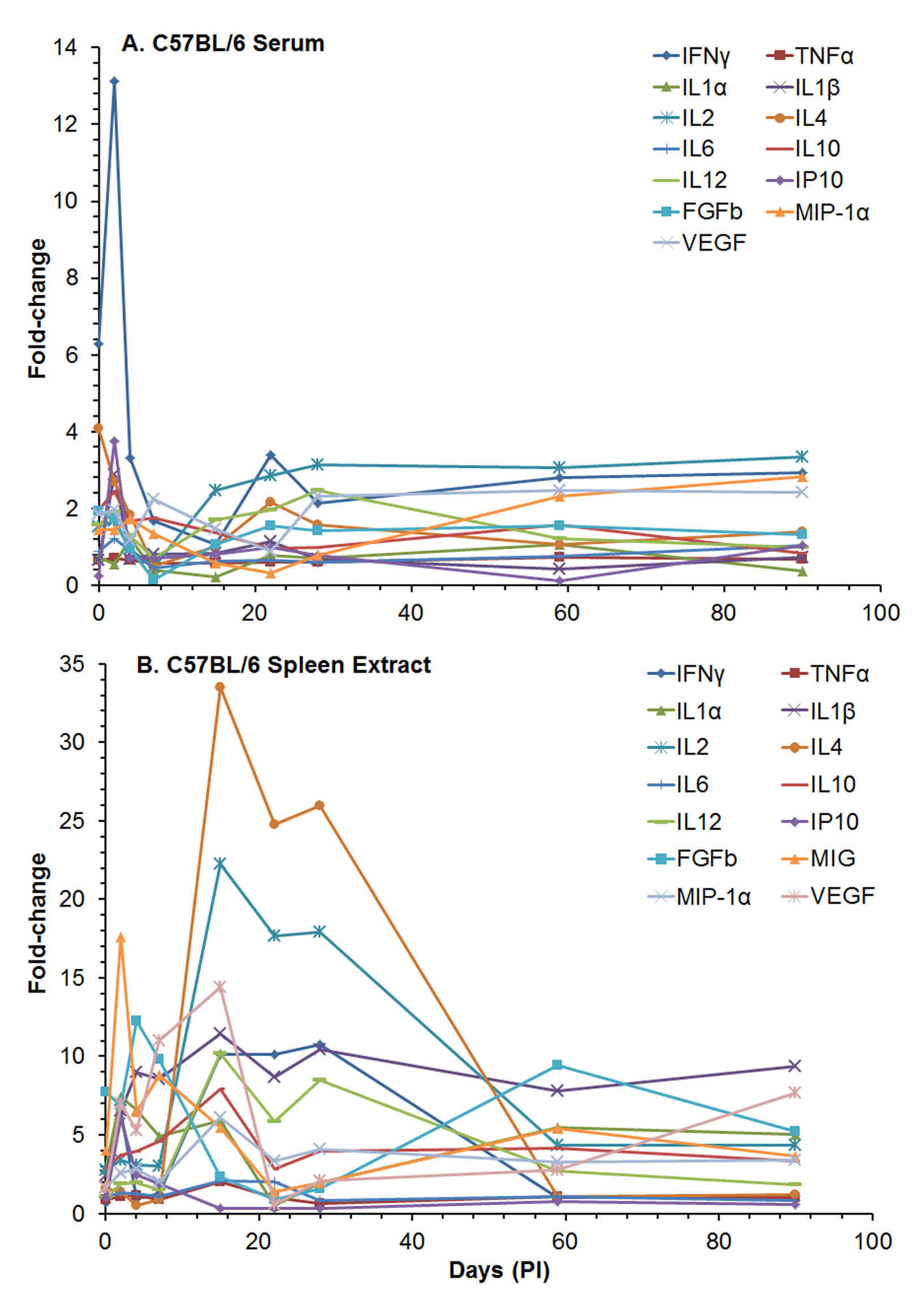

Supplement: S5 Fig — The amount of cytokines/chemokines present in sera (S7 Table) was determined. For changes in cytokine/chemokine levels in sera from C57BL/6 mice (A), we show changes in levels up to 91 days post-infection. We also show changes in cytokine/chemokine levels in spleen extracts for C57BL/6 mice (B) up to 91 days post-infection for comparison. For each mouse strain N = 5 at each time point. Fold-changes in cytokines/chemokines were determined by dividing the amount (pg/ml) present in sera of exposed mice (S7 Table) by the mount present in normal, naïve mice, where n was 10 for BALB/c and 4 for C57BL/6 mice. For C57BL/6 mice fold-change for MIG was not shown because it was very high (235-fold), and it would make it difficult to see the changes in the levels of the other cytokines/chemokines at the same time. (TIF) [file pone.0172627.s005.tif]
